# Promising Therapeutic Candidate for Myocardial Ischemia/Reperfusion Injury: What Are the Possible Mechanisms and Roles of Phytochemicals?

**DOI:** 10.3389/fcvm.2021.792592

**Published:** 2022-02-17

**Authors:** Cong Chen, Lin-Tong Yu, Bai-Ru Cheng, Jiang-Lin Xu, Yun Cai, Jia-Lin Jin, Ru-Li Feng, Long Xie, Xin-Yan Qu, Dong Li, Jing Liu, Yan Li, Xiao-Yun Cui, Jin-Jin Lu, Kun Zhou, Qian Lin, Jie Wan

**Affiliations:** ^1^Department of Cardiology, Dongzhimen Hospital Affiliated to Beijing University of Chinese Medicine, Beijing, China; ^2^Department of Cardiology, Xiyuan Hospital, China Academy of Chinese Medical Sciences, Beijing, China; ^3^Department of Cardiology, Dongfang Hospital Beijing University of Chinese Medicine, Beijing, China

**Keywords:** myocardial ischemia/reperfusion injury, phytochemicals, pharmacology, mechanisms, therapeutic implication

## Abstract

Percutaneous coronary intervention (PCI) is one of the most effective reperfusion strategies for acute myocardial infarction (AMI) despite myocardial ischemia/reperfusion (I/R) injury, causing one of the causes of most cardiomyocyte injuries and deaths. The pathological processes of myocardial I/R injury include apoptosis, autophagy, and irreversible cell death caused by calcium overload, oxidative stress, and inflammation. Eventually, myocardial I/R injury causes a spike of further cardiomyocyte injury that contributes to final infarct size (IS) and bound with hospitalization of heart failure as well as all-cause mortality within the following 12 months. Therefore, the addition of adjuvant intervention to improve myocardial salvage and cardiac function calls for further investigation. Phytochemicals are non-nutritive bioactive secondary compounds abundantly found in Chinese herbal medicine. Great effort has been put into phytochemicals because they are often in line with the expectations to improve myocardial I/R injury without compromising the clinical efficacy or to even produce synergy. We summarized the previous efforts, briefly outlined the mechanism of myocardial I/R injury, and focused on exploring the cardioprotective effects and potential mechanisms of all phytochemical types that have been investigated under myocardial I/R injury. Phytochemicals deserve to be utilized as promising therapeutic candidates for further development and research on combating myocardial I/R injury. Nevertheless, more studies are needed to provide a better understanding of the mechanism of myocardial I/R injury treatment using phytochemicals and possible side effects associated with this approach.

## Introduction

AMI remains the world's leading cause of morbidity and mortality (1). Of all the deaths, adverse acute ischemic events, such as ST-elevation myocardial infarction (STEMI), are the main triggers (2). In recent years, most of the endeavors in the processing of STEMI have been focused on guaranteeing the prompt coronary revascularization of the culprit artery and exploitation of pharmacological regimens for further preservation of the coronary blood flow (3, 4). Early primary percutaneous coronary intervention (pPCI; within 2 h since symptoms onset) has proved effective in reducing ischemia time to improve the outcomes of patients with STEMI (5); however, the cardiomyocytes begin to die having experienced a long-term ischemic environment. Even though reperfusion proves effective in limiting this process, it causes a spike of further cardiomyocyte injury (known as “reperfusion injury”) that contributes to final IS (6), which remains a crucial determinant of prognosis and is bound with hospitalization of heart failure as well as all-cause mortality within the following 12 months. While the ischemic injury increases with the severity and the duration of blood flow reduction, reperfusion injury achieves its maximum with a moderate amount of ischemic injury (7). Therefore, the addition of adjuvant intervention to limit cardiomyocyte death during myocardial I/R injury has become necessary.

The exact mechanisms of how the homeostasis of cardiac cells is impaired during myocardial I/R injury are not fully understood (8). Pathological changes, such as calcium overload, inflammation, apoptosis, neurohumoral activation, autophagy, and oxidative stress, are considered to be of equal contribution to I/R injury (9). Phytochemicals, the secondary metabolites and natural components of herbs, mainly composed of non-nutritive bioactive compounds, have long been recognized as promising therapeutic candidates for novel drugs (10). They are synthesized only in specific plant cells and do not take part in the energy metabolism nor the catabolic or anabolic ones (11). More than 10,000 phytochemicals have been discovered so far, including saponins, polyphenols, carotenoids, terpenes, and alkaloids, while many remain unknown (12). In recent years, they have attracted more attention as modulators of many cellular signaling pathways and by the ability of health improvement (13). For example, metabolic disorders, such as cardiovascular disease, cancer, and obesity, may benefit from many phytochemicals (14). Research and clinical studies have demonstrated the compounds' biological effects, such as antioxidant, anti-inflammatory, and cytotoxic activities, suggesting that these natural products may be potential to alleviate the myocardial I/R injury (15, 16).

This review provides a concise summary of the efforts of former researchers on how phytochemicals alleviate myocardial I/R injury and highlights the evidence of their cardioprotective-related mechanisms. We aim to present new insight into the development of potential treatments for myocardial I/R injury.

## The Mechanism of Myocardial I/R Injury

**Figure 1:**
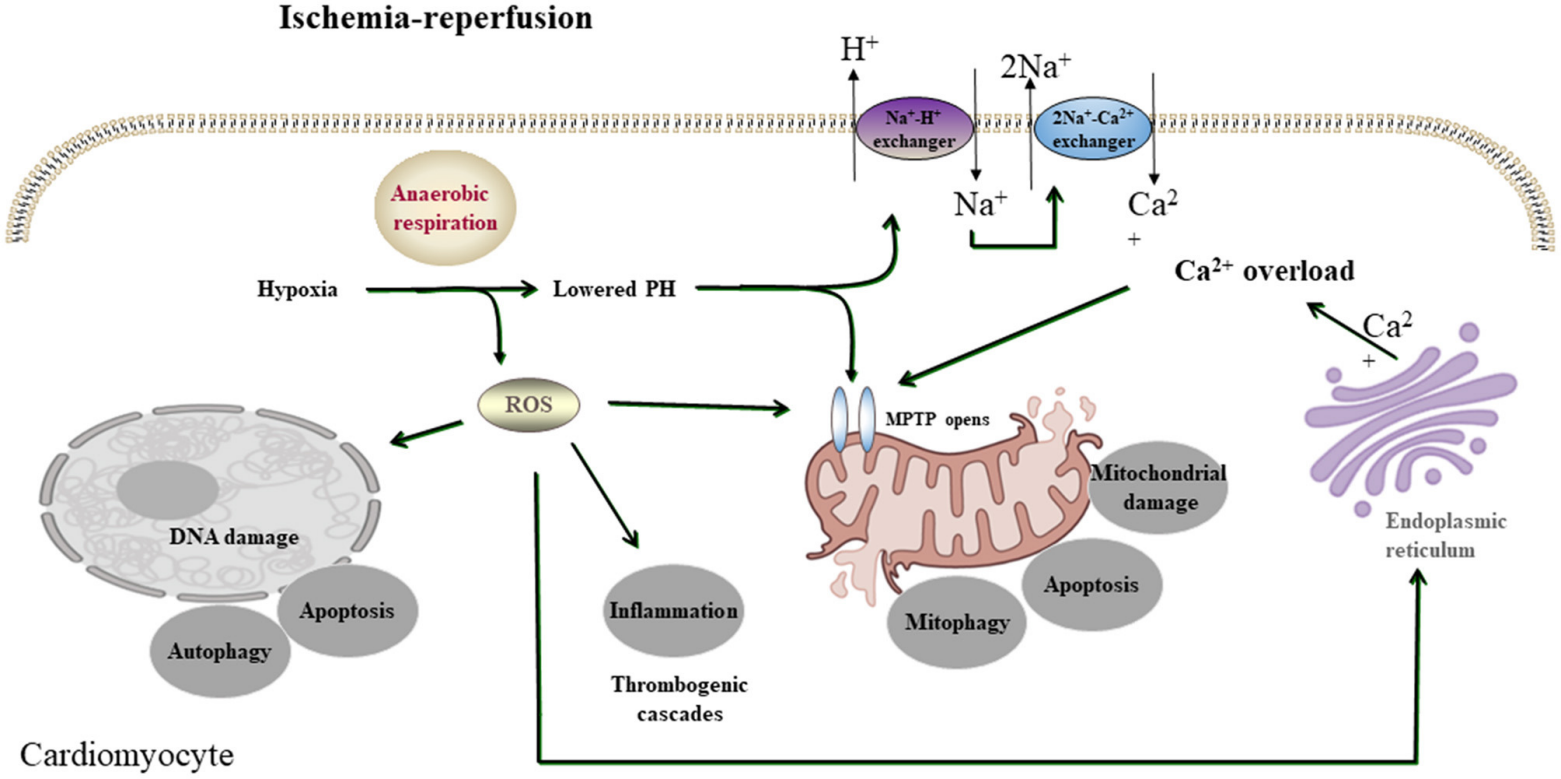
A simplified scheme of the mechanism of acute myocardial I/R injury. During acute myocardial ischemia, ischemic cardiomyocytes switch to anaerobic metabolism to provide ATP. However, this results in the Na+-H+ exchanger to extrude H+ and results in intracellular Na+ overload, which activates the 2Na+-Ca2+ exchanger to function in reverse to extrude Na+ and leads to intracellular Ca2+ overload. The endoplasmic reticulum also markedly reduces Ca2+ reuptake, which exacerbates intracellular Ca2+ overload. Ca2+ can also induce MPTP opening. During reperfusion, the influx of oxygen fuels production of ROS (oxygen paradox). Other sources of ROS include xanthine oxidase (endothelial cells) and NADPH oxidase (neutrophils). ROS can damage virtually every biomolecule found in cells, promote the opening of mPTPs, and activate inflammatory and thrombogenic cascades to exacerbate cell injury.

Many pathways that induce cell death, also known as apoptosis, programmed necrosis, or necroptosis, are initiated by myocardial I/R injury, involving several signaling pathways (9). Therefore, it is necessary to find improved protective strategies to prevent myocardial I/R injury, of which the related mechanisms have been widely studied. A growing number of pieces of research, both *in vitro* and *in vivo*, have proved phytochemicals to be potently cardioprotective on myocardial I/R injury, mainly by restraining irreversible cell death caused by apoptosis, autophagy, and necrosis *via* preventing calcium overload, oxidative stress, and inflammation (17) (Figure 1).

### Apoptosis

Apoptosis exists in several cellular organisms. Stimulation of apoptotic pathways leads to cell death in ischemic heart cells (18). Generally, the high level of reactive oxygen species (ROS) can be lethal for I/R-injured cells and attributed to cardiomyocyte apoptosis (19). It is proved that during myocardial I/R injury, apoptosis-related genes, such as signal transducer and activator of transcription 3(STAT3), B-cell lymphoma-C(BCL-C), and B-cell lymphoma-xL(Bcl-xL) in the myocardial tissue, are rearranged (20). There is evidence that the increased expression of these genes produces protective proteins against apoptotic pathways and reduces physiotherapy (21). Ca^2+^ signaling, which can be modulated and synchronized by mitochondria, is an essential part of apoptosis. Accumulation of Ca^2+^ in mitochondria leads to apoptosis (22). In porcine models of chronic myocardial ischemia and hibernation, autophagy-enhanced cardiomyocytes were negative for apoptosis, while apoptotic cells were negative for autophagy, suggesting that autophagy plays a protective role against apoptosis in this model (23). mTORC1 can sense cellular nutrient status (24) and inhibits myocardial I/R injury. Growth factor receptor-bound protein 1(GRb1) treatment antagonizes the inhibitory effect of mTORC1 (25). B-cell lymphoma-2(Bcl-2) has the potential to inhibit apoptosis, mitochondria disruption, the following cytochrome c(Cyt c) release, and, finally, caspase activation (26). Pretreating with Eupatilin can increase Bcl-2 expression, decrease BCL2-associated X(Bax), and cleaved caspase-3 expression induced by hypoxia-reoxygenation (H/R) in H9c2 cells (27).

### Autophagy

Autophagy is essential to maintain cellular homeostasis. But its effects on myocardial I/R injury are paradoxical (17). Autophagy is characterized by the formation of a cup-shaped pre-autophagosomal double-membrane structure, which surrounds cytoplasmic material and closes to form the autophagosome (19). Autophagosome clearance, which can cause autophagy acceleration and cardiomyocyte death, is damaged during I/R injury (28). The knocking out of Beclin1 heterozygous eliminates myocardial I/R-induced autophagosome formation, as well as reduces myocardial infarction and cell death (29). Likewise, 3-methyladenine (3-MA) reduces autophagy caused by I/R *via* prohibiting autophagy and increases survived cells (30). Hesperidin can inhibit excessive autophagy by triggering the PI3K/Akt/mammalian target of the rapamycin (mTOR) pathway. Hesperidin was found to be capable of reinforcing p-PI3K, p-Akt, and p-mTOR levels and downregulating LC3II and Beclin1, whereas its specific inhibitor, LY294002, obviously invalidated all the effects mentioned above (31).

On the contrary, autophagy has been widely reported to be beneficial to myocardial I/R injury. The recovery of myocardium function after I/R benefits from a high level of autophagy. However, the depletion of adenosine triphosphate (ATP) is possibly the reason for autophagosome-lysosome pathway impairment during ischemia, which correlates with permanent injury in contractile function (32). With enhanced autophagy, apoptosis decreases in cardiac myocytes, same as autophagy in apoptotic cells in the porcine model of chronic myocardial ischemia and hibernating myocardium. Therefore, there is a deduction that cells are protected by autophagy against apoptosis in this model (33). Also, glucose deprivation-mediated cell death can be promoted when autophagy is inhibited (29). It can be concluded that when autophagy is upregulated, cells are likely to survive during I/R. Resveratrol is found to alleviate I/R injury of the myocardium in diabetic patients by promoting programmed cell death and *via* upregulating Beclin 1 and LC3-II (34).

### Ca^2+^ Overload

When there is myocardium hypoperfusion, affected cardiomyocytes switch to use less oxygen, leading to lactate, H^+^, and nicotinamide adenine dinucleotide (NADH^+^) accumulation and cytosolic pH decrease. To reestablish acid-based balance, the plasmalemma Na^+^/Ca^2+^ exchanger is activated. Then, the extracellular H+ ions accumulated during ischemia raise the proton gradient across the plasmalemma and further result in cytosolic Ca^2+^ increase (9). In addition, under physiological conditions, inactive calpains compete with their endogenous inhibitor calpastatin and are stored in cellular cytosol (35). Calpain is activated by the elevation of intracellular calcium levels, and its conformational changes promote the intracellular translocation of Ca^2+^, where phospholipids close Ca^2+^ channels, activating downstream proteins or diminishing the Ca^2+^ threshold for calpain activation (36). Myocardial ischemia/reperfusion injury is associated with a calcium homeostasis imbalance (37). *In vivo* studies showed an increment in intracellular Ca^2+^ concentration caused by ischemia/reperfusion in isolated perfused mammalian hearts (36). Reperfusion leads to rapid alterations in ion flux and alters the state of ion exchange, resulting in intracellular calcium overload (38). Increased calcium overload plays a key role in apoptosis, cell cycle, and differentiation, modifying cardiomyocyte function.

### Oxidative Stress

Oxidative stress is the result of an imbalance between oxidants and anti-oxidants. When the blood supply in an ischemic area is reestablished, the influx of oxygen produces excessive ROS, which is harmful to the ischemic area. This phenomenon is called the oxygen paradox, meaning that reperfusion after ischemia can result in injury rather than protection. This is because ROS modifies the metabolism in cells and tissues, leading to dysfunction or even cell death (39). ROS is the reason why I/R is deleterious for cells and tissues (40). Thus, oxidative stress reduction may combat I/R injury, and further investigations are needed. NF-E2-related factor 2(Nrf2), a member of the NF-E2 family of nuclear basic leucine zipper transcription factors, promotes the detoxification of pro-oxidative stressors. The Nrf2 signaling pathway plays a critical role in protecting the ischemic myocardium from myocardial I/R injury. Nrf2 deficiency mice show increased oxidative stress as well as an aggravated cardiac injury during I/R (41).

### Inflammation

Inflammation is a strong shield to protect the body, but dysfunctional inflammation has much to do with the pathogenesis of many diseases. Leukocyte infiltration can be activated in the infarcted myocardial region *via* a complex inflammatory pathway to protect unaffected regions (42). Evidence shows that, in a heart, reperfused areas can be harmed by an excessively activated inflammatory reaction (43). Nuclear factor kappa-light-chain enhancer of the activated B cells (NF-κB) signaling pathway is crucial to cardiac I-R injury. NF-κB interacts with the nucleus by regulating more than 200 genes, among which some produce inflammatory cytokines, which ultimately lead to excessive inflammation. H9C2 cells, which are damaged by hypoxia through BRCA1/ROS-regulated NLRP3 inflammasome/IL-1β and NF-κB/TNF-α/IL-6 pathways, can be improved by Paeonol (44). When intercellular macromolecular proteins aggregate, they are called inflammasomes, which promote the maturation of inflammatory cytokines (45). NLRP3 consists of NLRP3, ASC, and caspase-1 precursor (Pro-Casp-1) (46) and is the most widely studied inflammatory pathway for now. Artemisinin can reduce the oxidative stress reaction due to its NLRP3-regulating ability (47).

## Cardioprotective Phytochemicals Attenuating Myocardial I/R Injury

We thoroughly illustrated the phytochemicals proved to possess protective effects in the heart against I/R injury. Because of their variety, phytochemicals have been classified into phenols, saponins, lignans, terpenes, alkaloids, quinones, polysaccharides, carotenoids, coumarin, and other compounds for a better summary in this review.

### Phenols

Phenolic compounds constitute one of the most ubiquitous groups of plant metabolites and are an integral part of both human and animal diets (48). Among numerous natural phytochemicals used to prevent myocardial I/R injury, phenolic compounds are particularly important because of their unique properties. Although, these compounds were first known for their antioxidant properties, several studies over the years have shown that they can exert protective effects against myocardial I/R injury. The mechanisms underlying these potential benefits include the regulation of different cell signaling pathways and gene expression.

#### Paeonol

Paeonol (2′-hydroxy-4′-methoxyacetophenone), isolated from the plant *Moutan Cortex*, was found to possess broad pharmacological effects on treating atherosclerotic lesions. This is associated with alleviating endothelial injury, ameliorating inflammation and oxidative stress, repressing platelet activation and aggregation, inhibiting vascular smooth muscle cell (VSMC) proliferation and migration, as well as lowering blood lipids (49–53). Pretreating with paeonol can significantly improve the hypoxia-reoxygenation (H/R) damage and the BRCA1 expression of H9C2 cells through the BRCA1/ROS-regulated NLRP3 inflammasome/IL-1β and NF-κB/TNF-α/IL-6 pathways. It may be a candidate drug for treating myocardial I/R injury (44).

#### Oridonin

Oridonin is a wide-studied flavonoid compound extracted from *Isodon rubescens (Hemsl.) H.Hara*, and it has a multitargeting anticancer effect (54). Lu et al. demonstrated that it exerts cardioprotective effects by reducing I/R-induced inflammatory injury. Pretreating with oridonin also reduced oxidative stress and downregulated the NLRP3 inflammasome pathway. These recent findings have shown the molecular mechanism of its alleviating myocardial I/R injury. Applying oridonin could help prevent and treat myocardial I/R injury (55).

#### Baicalin

Baicalin is a flavonoid compound isolated from the roots of *Scutellaria baicalensis Georgi*. It proves effective in treating diseases like cancer, osteoarthritis, hepatitis, and nephritis (56–58). It was reported that baicalin exerted antioxidant, anti-apoptotic, and anti-inflammatory properties (59). Studies demonstrated that baicalin was protective for rat cardiomyocytes through downregulating H/R-induced injury (60). It was demonstrated by Kong et al. that baicalin reduced I/R damage in the heart by its antioxidant and paracrine effects (61). Luan et al. demonstrated that by regulating the Akt/NF-κB signaling pathway, baicalin downregulated myocardial apoptosis and inflammation (62). Liu et al. reported that LV functions were improved and myocardial apoptosis was suppressed by baicalin *via* suppressing the CaSR/ERK1/2 signaling pathway in myocardial I/R injury rats (63). Xu et al. reported the protective effect of baicalin, *via* the JAK/STAT pathway, on myocardial I/R injury. In addition, baicalin reduced cardiomyocytes damage, downregulated cell death caused by I/R, and inhibited inflammation response in the heart by interfering with macrophages (64).

#### Resveratrol

The natural compound resveratrol was mainly extracted in fruits, such as peanut, grape, and berry. It has been demonstrated that resveratrol downregulates the pathological progression in many disease models, such as cancer or diabetes mellitus (65–67). Currently, resveratrol has been demonstrated to carry a potentially cardioprotective property against myocardial I/R injury *via* regulating inflammatory, angiogenesis, energy metabolism, mitochondrial function, and cardiomyocyte apoptosis (68, 69). Compared with vehicles, resveratrol significantly reduced the size heart infarction area in small animal studies both *in vivo* and *ex vivo*. Neither the reperfusion time nor the route of administration affects the effects of resveratrol (70). Resveratrol also exerts protection on myocardial post-I/R damage through inhibiting stromal interaction molecule1 (STIM1)-mediated store-operated Ca^2+^ accumulation (71), upregulating of Beclin-1 and LC-3II expression to induce autophagy (34) and regulating phosphorylation levels of proteins relative to the PI3K/Akt/e-NOS pathway (72). Concluding from the available data, resveratrol presents a significant limiting effect against myocardial I/R damage.

#### Polydatin

Polydatin, isolated from *Reynoutria japonica Houtt*., is another monocrystalline compound like resveratrol. The difference between them is at position C-3, where polydatin has the substitution of a glucoside group instead of a hydroxy group (73, 74). Polydatin exerts several pharmacological properties, such as anti-inflammatory, antioxidant (75), and alleviation of cardiac remodeling induced by pressure overload (74). Ling et al. reported that this compound aggravated autophagy and inhibited cell death during I/R or H/R, and co-treatment with adenovirus carrying short hairpin RNA for Beclin 1 and 3-MA, an autophagic inhibitor, would reverse this effect. Polydatin-treated mice showed a significantly reduced IS in heart tissue and a better heart function, compared with vehicle-treated mice, whereas these effects could be partly antagonized by 3-methyladenine (3-MA). These findings showed that polydatin treatment after infarction lowered myocardial I/R damage by enhancing autophagy to clear impaired mitochondria and to downregulate ROS and apoptosis (76).

#### Salvianolic Acid B

Salvianolic acid B (Sal B), derived from *Salvia miltiorrhiza Bunge*., is a water-soluble compound (77). Sal B exerts multiple effects, such as reducing inflammatory factor expression, inhibiting cell death, and alleviating oxidative stress (78, 79). Former evidence has shown that Sal B can alleviate oxidative stress, modulate calcium overload, promote endothelial function, stabilize mitochondrial membrane potential, and upregulate microRNA-30 (80), making it protective against myocardial I/R injury. A recent study has revealed that Sal B could alleviate myocardial I/R damage dose-dependently, promote cardiac function, decrease myocardial infarction size, reduce myocardial injury marker expression, inhibit inflammatory responses, increase PI3K/Akt expression, and decrease high-mobility group box protein 1(HMGB1) expression. The mechanism is that Sal B ameliorated myocardial I/R damage by promoting PI3K/Akt and decreasing the release of HMGB1 in rats (81).

#### 6-Gingerol

6-Gingerol (6-G), a main component of gingerols and a phenolic compound isolated from *Curcuma longa L*., exerts antioxidative, antiapoptotic, and anti-inflammatory effects (82). Sampath et al. demonstrated that 6-G could prevent atherosclerosis *via* reducing cell death caused by excess oxidative stress (83). El-Bakly et al. found 6-G significantly protected cardiomyocytes by inhibiting cell death *via* alleviating oxidative stress and doxorubicin-induced myocardial damage (84). Lv et al. reported that pretreating with 6-G remarkably promoted cardiac function and decreased IS and I/R-induced creatine kinase-MB levels. 6-G alleviates myocardial I/R injury by reducing I/R-induced cardiomyocyte cell death and upregulating the PI3K/Akt signaling pathway. This evidence proved that 6-G may be a candidate drug for alleviating myocardial I/R injury (85).

#### Oleuropein

Oleuropein, a glycoside compound, is of antispasmodic effects, and can also reverse arrhythmia. In the rabbit isolated heart, it increases the coronary blood flow by 50% (86). Oleuropein can also lower blood pressure as it strongly inhibits the angiotensin-converting enzyme, as a result of its highly reactive 2,3-dihydroxy glutaraldehyde structure. A study reported that oleuropein protected the heart from myocardial I/R injury. In a myocardial I/R rat model, oleuropein reduced CK-MB and lactate dehydrogenase (LDH) levels as well as infarction size in the heart. Oleuropein also inhibited the caspase-3 pathway and reduced p53, p-IκBα protein, p-extracellular signal-regulated protein kinase (ERK), and phosphorylated (p)-mitogen-activated protein kinase kinase (MEK) expression. This evidence proved that by regulating the MEK/ERK/STAT3 signaling pathway, oleuropein inhibits myocardial I/R in rats (86).

#### Calycosin-7-O-β-D-Glucoside

Calycosin-7-O-β-D-glucoside (CG) is a major isoflavone extracted from *Astragalus mongholicus Bunge*, which has been proved to exert anti-inflammatory (87) and antioxidant abilities (88). Studies demonstrated that CG could decrease the size of cerebral infarction in the process of cerebral I/R injury, maintain the stability of the blood-brain barrier, and reduce the I/R-induced neuronal injury (89). *In vitro* and *in vivo* experiments showed that CG activated the JAK2/STAT3 pathway and upregulated the secretion of IL-10, and, therefore, can protect cardiomyocytes from I/R-induced cell death (90).

#### Puerarin

Puerarin (7,4'-dihydroxy-8-C-glucosylisoflavone) is an isoflavone of broad pharmacological abilities (91), including treating cardiovascular and cerebrovascular diseases, which can be a potential drug in alleviating I/R injury (92). Puerarin lowers the lipid peroxidation level, and aldose reductase activity decreases superoxide ion radicals and protects endothelial cells (93). Studies showed that puerarin remarkably shrinks the myocardial infarction size and increased pressure in the left ventricular in rats with diabetes mellitus suffering from myocardial I/R. Puerarin significantly reduced oxidative stress, inflammation, and NF-κB protein expression. Furthermore, puerarin raised the levels of VEGFA and Ang-I, as well as increased nitric oxide (NO) production, caspase-3 activity, and phosphorylated-endothelial NO synthase protein expression. These findings illustrated that puerarin protected cardiomyocytes and served to reduce myocardial I/R damage (94).

#### Hesperidin

Widely found in citrus fruits, hesperidin is a flavanone glycoside with a molecular formula of C28H34O15 and a molecular weight of 610.57 Da (95). Hesperidin has been found to possess broad biological effects, including antioxidant, anti-cancer, radio-protective, anti-inflammatory, and anti-allergic, properties (96–99). Gandhi et al. reported that hesperidin reduced arrhythmias and apoptosis caused in myocardial I/R injury, also reduced inflammation and oxidative stress, decreased excessive autophagy, and promoted the PI3K/Akt/mTOR pathway (31, 100).

#### Luteolin

Luteolin is a flavone widely presented in several plants. Former experiments reported that Lut protected the cardiomyocytes from I/R damage by decreasing microRNA-208b-3p expression and inhibiting the PI3K/Akt pathway (101, 102), and partly reversing the low expression and activity of SERCA2a in the injured area (103). Other evidence demonstrated that it modulated SERCA2a by SUMOylation at lysine 585 (104). These studies demonstrated that luteolin prevents the heart from suffering from I/R injury.

#### Honokiol

Honokiol (HKL) is isolated from *Magnolia Officinalis Rehder and E.H. Wilson*, which has long been used as a herb in traditional Chinese medicine. It is known for its effect of treating various vascular diseases, including ischemia and infarction (105). HKL was reported to be able to alleviate cerebral I/R injury *via* relieving oxidative stress and downregulating inflammatory reaction (106). Early evidence also proved that HKL could limit the infarct area and reduce arrhythmia in rats with AMI (107), in which its antioxidative and antiapoptotic abilities played critical roles. What is more, HKL could also regulate the SIRT1/Nrf2 signaling pathway, which was also important for its cardioprotective effects (108). Tan et al. demonstrated that post-treating with HKL reduced myocardial I/R injury and promoted autophagic flux in C57BL/6 mice (109).

#### Tournefolic Acid B

Tournefolic acid B (TAB) is a newly discovered compound isolated from *Clinopodium chinense (Benth.) Kuntze*, a traditional Chinese herbal medicine with modern pharmacological effects, such as anti-inflammatory, antitumor, antiradiation, and lowering blood glucose. Yu et al. reported that TAB significantly prevented the heart from being damaged by I/R injuries by suppressing ER stress and oxidative stress through inhibiting PI3K/AKT pathways. *In vitro* and *ex vivo* experiments, both supported this conclusion, meaning that TAB likely inhibited cell apoptosis by resisting oxidation-endoplasmic reticulum stress *via* activating the PI3K/AKT pathway (110).

#### Orientin

Orientin, one of the major active flavonoids of *Persicaria orientalis (L.) Spach*, is a traditional Chinese herb. It was reported to exert broad pharmacological properties, including anti-oxidant, anti-inflammation, and anti-apoptosis (111). Former experiments have demonstrated that orientin protected myocardium from I/R damage probably by reducing cell death (112). Evidence showed that the protective effect of orientin against myocardial I/R damage is partly regulated through subtle induction of autophagy, which involves the AMPK-mTORC1 signaling pathway and the phosphorylation of Beclin 1/Bcl-2 interaction in ER (113).

#### Icariin

Icariin, a natural flavonoid glucoside, is of broad pharmacological properties (114). Studies proved that icariin had antioxidant, antidepressant, anti-inflammatory, neuroprotective, and male sexual function improvement effects *in vitro* (115–119). In congestive heart failure rats, icariin promoted left ventricular function and attenuated cardiac remodeling *via* down-regulating matrix metalloproteinase-2 and−9 activity and inhibited cardiomyocyte death (119). Previous experiments have proved that myocardial function was protected by icariin from myocardial I/R damage in rats. It reduced IS, decreased I/R injury, and inhibited its remodeling. These properties of icariin are associated with lower blood indicators CK, IMA, and LDH levels in the serum and upregulated PI3K/Akt/eNOS pathway in rats' ischemic tissue, making it a candidate drug for preventing and resisting I/R injury in the early stage (120).

#### Curcumin

Curcumin [1,7-bis(4-hydroxy-3-methoxyphenyl)-1,6-heptadiene-3,5-dione], a natural compound isolated from the roots of *Curcuma longa L*., exerts wide pharmacological activities, including antioxidant, anti-inflammatory, and anticarcinogenic abilities in several rodent models. Previous experiments have suggested that curcumin is protective against some cardiovascular pathological conditions leading to heart failure (121, 122). Curcumin can improve heart function and ameliorate heart damage because it reduces oxidative stress and cell death, specifically by activating the phosphorylation of JAK2 and STAT3, increasing the myocardium Bcl-2/Bax expression and inhibiting caspase-3 (123).

#### Salvianolic Acid A

Salvianolic acid A is a water-soluble compound of *Salvia miltiorrhiza Bunge*, which is known to exert broad effects, including antioxidant, anticarcinogenic, anti-fibrotic, anti-inflammatory, and anti-platelet aggregation (124, 125). A previous study reported that, in diabetic rats, the JNK/PI3K/Akt signaling pathway correlated with myocardial I/R injury, and Sal A improved the recovery of heart function and prevented cell death following I/R damage in this model. This study provided critical evidence of the molecular mechanisms relating to Sal A's cardioprotective effects on I/R-injured diabetic rats (126).

#### Astilbin

Astilbin, a flavonoid compound extracted from the roots of *Smilax china L*., which has been long used in the traditional Chinese medicine clinical practice, has been found to have anti-hepatic, anti-arthritic, and anti-renal injury effects (127–129). Researchers have reported that in the early stages of STZ-induced diabetes rats, Astilbin resulted in a better heart function recovery caused by myocardial I/R damage *via* constraining inflammation and reducing HMGB1, phosphorylating NF-κB in ischemic myocardial tissue (130).

#### Eupatilin

Eupatilin (5,7-dihydroxy-3′,4′,6-trimethoxyflavone), which comes from the species of Artemisia plants, is a flavonoid of bioactive properties. Increasing studies have demonstrated that eupatilin exerts anti-allergic, anti-oxidant, anti-tumor, and anti-inflammatory activities (131–133). Experiments proved that eupatilin alleviated myocardial I/R damage *via* decreasing ROS and cell death by activating the Akt/glycogen synthase kinase-3β(GSK-3β) signaling pathway. Eupatilin is of therapeutic usage in treating myocardial I/R injury (27).

#### Syringic Acid

Syringic acid (SA), a natural O-methylated trihydroxy benzoic acid isolated from *Dendrobium nobile Lindl*., possesses broad biological activities, such as anti-oxidant, anti-tumor, and anti-inflammatory properties (134, 135). SA was found to prevent I/R injury. Tokmak et al. (136) reported that pretreating with SA in the spinal cord could reduce oxidative stress and neuronal degeneration induced by I/R. SA also ameliorated renal I/R injury (122). Liu et al. verified that SA exerted cardioprotective activities against myocardial I/R damage *via* activating the PI3K/Akt/GSK-3β signaling pathway and inhibiting the mitochondria-induced cell death (137).

#### Epigallocatechin-3-Gallate

Epigallocatechin-3-gallate (EGCG), the most widely studied catechin extracted from green tea leaves, was reported to reduce cardiovascular risk (138) through anti-inflammation and antioxidant activities, lowering serum cholesterol levels and reducing atherosclerosis (139, 140). Also, EGCG pretreatment limits IS caused by ischemia in the rat heart (141). Studies verified that giving rats EGCG together with reperfusion protected their hearts from regional myocardial I/R damage by activating pro-survival kinases, involving PI3K-Akt/GSK-3β and inhibiting cell death pathway p38 and JNK but not involving the ERK pathway (142).

#### Icariin

Icariin was found in *Epimedium brevicornu Maxim*., and it is a major compound of the herb Yin Yang Huo in traditional Chinese medicine. Its effects include anti-inflammation, antidepression, and antineoplastic properties. It also improves male sexual function, enhances bone healing, and protects neurons (143). Icariin postconditioning could attenuate myocardial I/R injury in the experimental rat model by activating the PI3K/Akt pathway and reducing cell death (144).

#### Troxerutin

Troxerutin, also known as vitamin P4, a derived natural bioflavonoid, owns wide biological properties including anti-oxidation and anti-inflammation (145). Pretreatment with this flavonoid extracted from Sophora japonica and Dimorphandra gardneriana (146) could decrease the occurrence of arrhythmias induced by I/R in diabetic and healthy rat hearts. Studies proved that the possible mechanism of its cardioprotective abilities may be the downregulating of inflammatory cytokines and inflammatory reactions in the heart (147).

#### Tilianin

Tilianin proves effective in upregulating NO synthase expression and NO production. It also acts as an anti-inflammatory ingredient (148). A study demonstrated that Tilianin exerted a significant protective effect on myocardial I/R-injured rat hearts (149). Studies verified that Tilianin pretreatment ameliorated the myocardial infarction and I/R damage in rats *via* the preservation of mitochondrial functions. The underlying mechanisms of Tilianin's cardioprotective activities may be mitochondrial preservation and cell apoptosis inhibition (150).

#### Isoquercitrin

Isoquercitrin is a natural compound present in vegetables, herbs, and flowers (151). It has been discovered that isoquercitrin reduces inflammatory, allergic reactions, and oxidative stress. (152). Isoquercetin was reported to reserve mitochondrial function and inhibit Cyt release induced by I/R injury in H9C2 cells (153). These findings verified that isoquercetin can be a candidate drug with cardiovascular protective effects in the treatment of myocardial I/R injury.

#### Vitexin

Vitexin (apigenin-8-C-β-D-glucopyranoside) is a flavonoid derived from *Acer rubrum L., Anthurium andraeanum*, and *Cucumis sativus L*. (154). Early studies have shown the hypotensive property and anti-inflammatory ability of vitexin. Recent pieces of research demonstrated vitexin's potential application for treating diseases like cancer (155). *In vivo* studies verified that vitexin was protective against myocardial I/R damage in rat heart, of which the mechanism could be related to its antioxidation ability and lowering of the levels of inflammatory cytokines by inhibiting the expression of NF-κB and TNF-α, as well as the upregulating phospho-ERK and downregulating phospho-c-Jun expression (156).

#### Apigenin

As a member of the non-mutagenic flavone subclass, Apigenin, isolated from the leaves of *Apium graveolens L*., exhibits low levels of toxicity. Previous studies have revealed Api's broad bioactive effects, including antiviral, antibacterial, anti-carcinogenic, antioxidant, and anti-inflammatory (157). Studies showed that Api could inhibit the p38 MAPK signaling pathway to protect the myocardium from I/R damage (158).

#### Bauhinia Championii Flavone

Bauhinia championii flavone (BCF) is extracted from the stem of *Phanera championii Benth*., of which the extract promotes blood circulation, reduces inflammatory and oxidative stress, and prevents platelet aggregation (159). Jian et al. reported BCF's protective properties of myocardium suffering from I/R damage in rats. The underlying mechanisms may depend on its ability to inhibit lipid peroxidation and activate the anti-oxidative system, its anti-inflammatory property by downregulating inflammatory levels by inhibiting signaling pathways, such as TLR4/NF-κB. It could also inhibit Bax/Bcl-2 ratios and caspase-3 activation (160).

#### Gastrodin

The phenolic glycoside gastrodin (GAS) is a monomeric component derived from *Gastrodia elata Blume* and has a variety of properties. It has long been used in treating cerebrovascular and cardiovascular diseases (161). Previous studies have demonstrated that GAS could reduce oxidative stress, lower inflammatory levels, and elevate hypoxia tolerance (162). Neighboring mitochondria and cardiomyocytes could be protected by the pretreatment of GAS *via* promoting autophagic flux and eliminating dysfunctional mitochondria (163).

#### Pinocembrin

The flavonoid pinocembrin, mainly found in *Propolis*, possesses antibacterial, anti-oxidant, and anti-inflammatory properties (164, 165). Pretreating with pinocembrin reduced cardiac arrhythmia in I/R rats through the enhancement of Na^+^-K^+^-ATPase and Ca^2+^-Mg^2+^-ATPase and the upregulation of Cx43 and Kir2.1 protein expression levels. *Via* upregulating gap junction- or ion channel-related gene or protein expression, cellular gap junction connexin function and IK1 current were restored, and cardiac arrhythmia was suppressed by correcting the P-R intermittent period, QRS duration, intracellular transmission velocity (166).

#### Silibinin

Silibinin, a polyphenolic flavonoid, is the main active component extracted from the seed of silybum marianum (milk thistle) or artichoke (cynara scolymus) (167). Previous studies have reported silibinin confers protective advantage in improving both liver and cerebral function after I/R, which raises concern about the role of silibinin against reperfusion injury in other tissues, especially in myocardium (168, 169). Chen et al. demonstrated silibinin reduces cardiomyocytes apoptosis, attenuates mitochondrial impairment and endoplasmic reticulum (ER) stress, alleviates ROS generation, neutrophil infiltration, and cytokines release (170). Furthermore, silibinin plus BAY 11-7082 (a selected NF-κB inhibitor) do not provide incremental benefits in improving myocytes apoptosis, oxidative stress, and inflammation in comparison with NF-κB signaling inhibition only. Thus, silibinin could prevent myocardial I/R injury by inhibiting cardiomyocytes apoptosis, reducing ER stress and oxidative stress, and modulating inflammatory response *via* deactivation of the NF-κB signaling pathway.

### Saponins

Saponins are known as surface-active compounds that are widely distributed in the plant kingdom (171). They comprise a non-polar aglycone or non-saccharide moiety, coupled with polar mono or oligosaccharides. Saponins mainly include four-ring triterpene saponins and five-ring triterpene saponins. In recent years, many studies have shown that the saponins extracted from herbal are great protective of myocardial I/R injury *in vivo* and *in vitro*. The mechanisms are diverse and mainly involve regulating energy metabolism and calcium homeostasis, and inhibiting oxidative stress and inflammation (172).

#### Polyphyllin I

Polyphyllin I (PPI) is a steroidal saponin extracted from the roots of *Paris polyphylla*. PPI's anti-cancer effect *via* inhibiting the proliferation and growth of tumor cells has been proved in previous studies, making it an anti-cancer drug candidate (173–175). A recent study has shown that PPI could prevent myocardial I/R injury, decrease myocardial death, and reduce the inflammation response and oxidative stress after I/R. Also, PPI activates NF-κB p65. Therefore, it can be deduced that PPI is protective of myocardial I/R injury in rats (176).

#### Ginsenoside Rb1

Ginseng (*Panax ginseng C.A.Mey*.) can improve the immune system (177). In many cases, Ginsenoside Rb1 (GRb1) represents ginseng saponins of Panax ginseng C. A. Mey. Recent animal studies have shown that GRb1 is protective of many myocardial diseases, including myocardial I/R injury (178). *In vivo* and *ex vivo* studies have demonstrated GRb1 reduced myocardial I/R injury, and the underlying mechanism is by reducing both CK-MB and Trop l levels after I/R. GRb1 improves cardiac function as well as increases Bcl-2 expression by activating the phosphorylation of mTOR, inhibiting apoptosis-related proteins Bax and cleaved-caspase 3 (25).

#### Gypenoside

Chinese doctors have been using G. pentaphyllum [*Gynostemma pentaphyllum* (*Thunb*.) Makino] to treat diseases for hundreds of years (179). The main component of G. pentaphyllum is gypenoside (GP), which is known for its antitumor, anti-inflammatory, and anti-oxidative effects (180, 181). To our interest, GP also exhibits protective property against I/R injury. Qi et al. proved that GP protected I/R-injured cerebral neuronal (182). Chang et al. reported the mechanism of this protective effect, which is by lowering the miR-143-3p level through the activation of the AMPK/Foxo1 pathway. The changes in signaling pathways eventually resulted in the improving condition of I/R-induced OGD/R in H9c2 cells in rat myocardial tissue. No studies before had unveiled gypenosides' effect on miR functions, which means this new finding could be used as a novel strategy for myocardial I/R injuries (183).

#### Astragaloside IV

As one of the main active components extracted from *Astragalus membranaceus Bunge*., the lanolin alcohol-shaped Astragaloside IV (AS-IV) is a tetracyclic triterpenoid saponin with high polarity, which has antiapoptotic effects. It has been demonstrated that AS-IV possessed anti-ischemic properties against cerebral I/R injury, pulmonary and cardiovascular disease, diabetic nephropathy, and liver fibrosis (184, 185). The pharmacologic effects of AS-IV include regulating the calcium balance, antioxidative stress, anti-inflammatory, anti-apoptosis, antifibrotic, anti-diabetes, immunoregulation, and cardioprotective effect *via* different signaling pathways (184–186). A review of AS-IV on animal studies demonstrated that AS-IV's cardioprotective effect of antioxidant, anti-apoptosis, and anti-inflammatory in acute myocardial I/R injury depended largely on improving the circulation and upregulation of angiogenesis (187). A previous study reported that AsIV treatment attenuated myocardial I/R injury *via* inhibition of Toll-like receptor 4- and nuclear factor-κB-mediated inflammatory responses and subsequent myocardial apoptosis in a rat model (188). Further study demonstrated that AsIV treatment attenuated myocardial injury, reduced cardiomyocyte apoptosis, decreased [Ca^2+^]_i_, inhibited CaSR expression, and increased ERK1/2 phosphorylation levels. These findings not only provided the underlying mechanisms of the cardioprotective effect of AsIV but also further demonstrated the pivotal role of CaSR in myocardial I/R injury (189).

#### Ginsenoside Rg3

Ginsenoside Rg3 can improve cardiac functions by mitochondria dynamic remodeling and increasing the number of mitochondria (190). It can also attenuate myocardial I/R injury by regulating Akt/endothelial NO synthase (191). Ginsenoside Rg3 exhibits anti-apoptosis and anti-inflammation properties, which is the underlying mechanism of heart function impairment induced by I/R (192).

#### Ginsenoside Rb3

Ginsenoside Rb3, a component isolated from Panax ginseng (*Panax ginseng C. A. Meyer*), is drawing increasing attention in the treatment of cardiovascular diseases, including myocardial I/R injury. It has been found out that ginsenoside Rb3 exhibited a protective effect on neurons on the I/R injury model *in vitro* by inhibiting cell death and inflammatory cytokines (193). It was found by Ma et al. that ginsenoside Rb3's protective effect partly depended on inhibiting the NF-κB pathway, meaning that ginsenoside Rb3 can be a potential treatment for myocardial I/R injury (194).

#### Platycodin D

Platycodin D is among the main saponins of *Platycodon grandiflorus* (195). It possesses a variety of effects, including antiinflammation, anti-atherosclerotic, and anti-oxidant (196). Studies showed that Platycodin D could protect the heart from H/R-induced, Akt/Nrf2/HO-1 pathway-mediated oxidative stress, cell damage, as well as cell apoptosis (197).

### Lignans

Lignans are a large class of natural compounds comprising two phenyl propane units. Lignans have been found rich in fruits, seeds, and vegetables, and received widespread interest due to their various biological activities, including antioxidant, antitumor, antibacterial, antiviral, insecticidal, fungistatic, estrogenic, and antiestrogenic activities (198).

#### Isovaleroylbinankadsurin A

Isovaleroylbinankadsurin A (ISBA) is a dibenzocyclooctadiene lignan extracted from S*chisandra Chinensis (Turcz.) Baill*. (199). ISBA possesses more than anti-inflammatory, anti-oxidant, and anti-tumor abilities (200, 201). It was reported that ISBA protected I/R-injured cardiomyocytes in models both *in vitro* and *in vivo*. Apoptosis induced by H/R injury was significantly inhibited *via* the mitochondrial-dependent pathway by ISBA. ISBA's protective effect on cardiomyocytes was mainly by activating the reperfusion injury salvage kinase (RISK) pathway. What is more, ISBA remarkably promoted the cellular anti-oxidative capacity by activating the RISK pathway, and, therefore, reduced oxidative damage induced by I/R injury by inhibiting the ROS generation, which proved ISBA's potential to be as a candidate drug for cardiovascular diseases (202).

#### Schisandrin B

Schisandrin B (Sch B) is also derived from the fruit of *Schisandra chinensis (Turcz.) Baill*., a common herb of traditional Chinese medicine (203). Sch B exerts hepatoprotective, anticancer, antioxidant, and antiinflammatory abilities (204). Zhang et al. reported that the pretreatment of Sch B on cardiomyocytes could decrease the size of the infarct area, promote the antioxidant ability, inhibit the ERS-induced apoptosis, and protect the myocardium (205).

#### Sauchinone

Sauchinone is also extracted from *Schisandra Chinensis (Turcz.) Baill*. (206), which protects cardiomyocytes from I/R injury in rats. Previous studies have shown it can inhibit p38, JNK, and other cell apoptosis signaling pathways (207).

### Terpenes

Terpenes represent one of the largest groups of plant secondary metabolites, with ~55,000 different structures (208). Depending on the number of linked isoprene units, the resulting terpenes are classified into hemi-, mono-, sesqui-, di-, sester-, tri-, sesquar-, tetra-, and polyterpenes. For many decades, it has been suggested that terpenes and terpenoids are potential chemopreventive and therapeutic agents for various diseases (209).

#### Glaucocalyxin A

Glaucocalyxin A (GLA) is derived from *Isodon japonicus var. glaucocalyx* (*Maxim*.). *H.W.Li* exerts wide bioactive effects, including inhibiting platelet aggregation (210), suppressing the immune system, protecting DNA damage, and cytotoxic activity (211). Previous studies have demonstrated that GLA restored heart function, decreased infarction size, and inhibited apoptosis signaling pathways in mice hearts injured by myocardial I/R. These abilities of GLA may associate with its anti-platelet effect and the reduction of microvascular thrombosis. With decreased bleeding risk, GLA may be a potential therapy for alleviating myocardial RI during cardiac revascularization (212). Another study demonstrated that by activating the Akt/Nrf2/HO-1 pathway, GLA protected H9c2 cells against H/R-induced damage. Therefore, GLA might be a candidate drug for preventing and treating MI (213).

#### Artemisinin

Artemisinin, isolated mainly from *Artemisia annua L*., is a sesquiterpene lactone compound with a peroxisome bridging group structure. Recent pieces of research have reported that it was not only anti-malaria but also an anti-tumor agent. Artemisinin can promote cell death, block cell cycle, prevent angiogenesis, and tumor metastasis (214). Its protective effect against I/R injury is mainly due to the activation of the NLRP3 inflammasome pathway, but preconditioning with artemisinin preconditioning could significantly suppress NLRP3 inflammasome activation. On the rat I/R injury model, artemisinin could reduce ROS and inflammation induced by I/R injury, therefore promoting myocardial recovery, including reducing the size of myocardial infarction and inhibiting cardiomyocyte apoptosis and autophagy (47).

#### Geniposide

Geniposide (C17H24O10, GP), one of the major components of the fruit of *Gardenia jasminoides J. Ellis* and is found in nearly 40 species of herbal plants, is also a well-studied iridoid glycoside (215). There have been a growing number of studies on the bioeffects of geniposide over the past few decades. It has been proved to be antidiabetic, antioxidant, antithrombotic, analgesic, hepatoprotective, neuroprotective, anti-inflammatory, antidepressant, cardioprotective, immune-regulatory, and antitumoral (216). It reduced the myocardial infarct area and apoptosis and promoted heart function. *In vitro* studies demonstrated, in H9c2 cells, GP enhanced the cell viability and prevented apoptosis during H/R. Both *in vivo* and *in vitro* experiments demonstrated that GP downregulated the expression of proteins related to autophagy and prevented autophagosome accumulation. Rapamycin administration could reverse these effects. In summary, GP protected cardiomyocytes from I/R damage and inhibited autophagy by activating AKT/mTOR signaling pathways (217).

#### Ginkgolide B

Isolated from the leaves of Ginkgo, Ginkgolide B (GB) is a diterpene lactone compound and has a strong effect on inhibiting platelet aggregation (218). Its anti-inflammatory, antioxidant, and anti-apoptotic properties have made it protective of stroke, both ischemic and hemorrhagic (219–221). In hydrogen peroxide-treated H9c2 cells, they pretreated with GB-activated PI3K/Akt/mTOR signaling pathway and upregulated the phosphorylation levels of Akt and mTOR and, therefore, inhibited cell apoptosis (222). *Via* activating the A20-dependent NF-κB signal pathway, GB could also ameliorate I/R-induced inflammatory damage both *in vivo* and *in vitro* (223). Thus, GB protected against myocardial I/R injury by inhibiting ER stress-induced apoptosis *via* the PI3K/AKT/mTOR signaling pathway. This finding suggests GB may be a promising therapy in treating I/R injury (224).

#### Araloside C

Araloside C is among the major triterpenoid compounds derived from *A. elata* and was found to significantly promote heart function (225). Araloside C was proved to protect the myocardium from I/R damage by inhibiting ROS generation as well as Ca2+ overload. It is demonstrated that such cardioprotective effect is due to its ability to combine with the Hsp90 protein and interact with the ATP/ADP-binding domain of Hsp90 (226).

#### Triptolide

Isolated from *Tripterygium wilfordii Hook.f*., triptolide possesses neuroprotective, anti-tumor, and anti-inflammatory abilities (227). Triptolide can alleviate cerebral and hepatic I/R injuries in experiments (228, 229), and it could promote heart condition and reduce inflammation and oxidative stress induced by I/R in rats. Such protective effects of the heart may relate to triptolide's influences on the Nrf2/HO-1 defense pathway (230).

### Alkaloids

Alkaloids, a class of nitrogen-containing basic organic compounds found in nature, are varied and complex. Alkaloids are mainly plants, but some are also found in animals. Alkaloids have an extensive pharmacological function. These significant biological activities often play a therapeutic role in Chinese herbal medicine management.

#### Berberine

Derived from several medicinal plants, such as *Coptis Chinensis Franch*. and *Berberis vulgaris L*., berberine (BBR) is an alkaloid with broad bioactive effects and has been used for treating many diseases (231). It showed antiapoptotic and antiinflammatory abilities both in cell and animal experiments (232). Huang et al. reported it enhanced H/R-induced cell viability and reduced I/R-induced IS and autophagy in cardiomyocytes (233). Also, BBR decreased CK-MB, LDH, and cTnI serum levels by decreasing myocardial cell death and promoting mitochondrial functions (234). Besides, BBR protects neurons by modulating cell death (235). It protects the myocardium from myocardial I/R injury *via* promoting proliferation, attenuating apoptosis *via* the mitophagy-mediated HIF-1α/BNIP3 pathway (236).

#### Galanthamine

Galanthamine protects neurons *via* activating the cholinergic pathway in the heart to prevent ischemic injury (237) and is important in promoting heart fitness and limiting IS (238). A previous study reported that it reduced cardiac dysfunction and alleviated endoplasmic reticulum stress (ERS)-related cell death induced by I/R *via* downregulating the expression of CHOP, Cleaved caspase 12, and caspase 3, as well as upregulating the expression of CADD34 and BiP in rats. It could also mitigate I/R-induced myocardial fibrosis in rats by inhibiting the expression of α-SMA and Collagen I. It was demonstrated that the mechanism of its cardioprotective and apoptosis-inhibiting effects were suppressing AMPK/Nrf2 pathways (239).

#### Matrine

The quinolizidine alkaloid compound Matrine, extracted from *Sophora flavescens Aiton*, which has been used as a herb in China, possesses antivirus, antitumor, antiallergic, anti-inflammatory, and antifibrotic effects (240–243). It activates the JAK2/STAT3 pathway and the upregulation of HSP70 expression. Guo et al. reported that the compound remarkably increased cell viability suppressed by H/R by decreasing lactate dehydrogenase and inhibiting creatine kinase activity *in vitro*. Also, it was proven to reduce CK-MB and TnI levels in the blood and decrease the size of the infarcted area in the heart as well as the I/R-induced apoptotic index of cardiomyocytes *in vivo*. It alleviated myocardial I/R damage by increasing HSP70 expression *via* activating the JAK2/STAT3 signaling pathway (244).

#### Palmatine

Palmatine, a natural quaternary protoberberine in the class of isoquinoline alkaloids, possesses pharmacological effects, such as anti-inflammatory and antioxidant (245–247). A previous study demonstrated that it could protect cardiomyocytes from I/R damage in rats. Its possible mechanism is relieving oxidative stress and regulating inflammatory mediators (248).

#### Capsaicin

Capsaicin is extracted from capsicum plants, such as *Capsicum annuum L*. and is widely used in food, medicine, and pharmacy (249). Pretreating with this compound protects H9c2 cells from H/R injury by upregulating 14-3-3η expression, modulating Bcl-2 and Bax expression and activity, reducing ROS generation, limiting mPTP opening, inhibiting caspase-3 activity, and, ultimately, suppressing cardiomyocytes apoptosis (250).

### Quinones

Quinones are mainly divided into four types: benzoquinone, naphthoquinone, phenanthraquinone, and anthraquinone. They naturally occur in bacteria, fungi, animals, and plants and have a variety of pharmacological effects. They are produced in organisms and are utilized as electron-transfer agents, pigments, and in defense mechanisms (251).

#### Sodium Tanshinone IIA Sulfonate

Among the derivatives of tanshinone, IIA is sodium tanshinone IIA sulfonate (STS), a major lipophilic constitute of *Salvia miltiorrhiza Bge* (252). Studies have shown that it was cardioprotective against several cardiovascular diseases and neuroprotective against neural dysfunction (253–255). Previous studies demonstrated that it also showed pharmacological actions including anti-oxidative stress and anti-inflammation (256, 257). The study provides some evidence that this compound was significantly protective in treating myocardial I/R injury in rats. Its antioxidant ability partly improves heart condition (258).

#### Shikonin

Shikonin is isolated from *Lithospermum erythrorhizon Siebold and Zucc*., which has been used for treating several inflammatories and infectious conditions (259). It has been reported that it exerted anti-inflammatory, antibacterial, antiviral, and antioxidant activities (260). The potential merits of pretreating it in H/R-induced cardiomyocyte apoptosis are partly regulated through activating the PI3K/Akt signaling pathway (261).

### Polysaccharides

Polysaccharides are widely distributed as natural ingredients in vegetables and fruits. Several studies have shown that the polysaccharides improve cardiovascular diseases through various mechanisms, such as anti-oxidative stress, regulating metabolism, anti-inflammatory, anti-cancer, and immunity-booster properties (262).

#### Fucoidan

Fucoidan is a sulfated polysaccharide molecule that has been known for its anticancer abilities. It is isolated mainly from the cell wall of different species of brown algae (*Phaeophyta*), a varied group of organisms (263). Omata et al. reported that, in the rat myocardial I/R injury model, there is a limited myocardial-IS and inhibited neutrophil accumulation, and one of the possible mechanisms could be the blockade of P-selectin-mediated neutrophil rolling on the vessel wall (264). Li et al. reported fucoidan improved left ventricular systolic pressure (LVSP), left ventricular end-diastolic pressure (LVEDP), and the contractility index in the rat myocardial I/R injury model, and could regulate the inflammation response *via* HMGB1 and NF-κB inactivation in I/R-induced myocardial damage (265).

### Carotenoids

Carotenoids are naturally found in the natural ingredients, particularly in fruits, vegetables, and algae. At present, more than 750 kinds of carotenoids have been identified, of which there are about 100 in edible foods. Carotenoids exhibit several biological and pharmaceutical benefits, such as anti-inflammatory, anti-cancer, and immunity-booster properties (266).

#### Lycopene

Lycopene is a natural compound whose antioxidant effects have been widely studied (267). Previous studies demonstrated that low levels of circulating lycopene are related to a higher risk of cardiovascular diseases (268, 269). It was proved that pretreatment with 1-μM lycopene before reoxygenation remarkably decreased cardiomyocytes apoptosis induced by H/R. Moreover, IV injection of 1-μM circulating lycopene significantly decreased the risk of MI during *in vivo* I/R in mice and effectively inhibited the oxidation of fatty acid and the activation of JNK signaling during reperfusion (270).

#### Retinol Palmitate

Retinol palmitate, an analog of vitamin A, exhibits effective peroxyl radical scavengers *via* suppressing peroxidation (271). It has been proved to exert the ability to promote neuronal differentiation, neural patterning, and axonal growth, making it potentially neuroprotective against cerebral I/R damage (272). It could also limit myocardial IS and inhibit cellular apoptosis *via* the downregulation of proapoptotic-related proteins expression and the upregulation of SOD-related proteins expression. Tao et al. suggested that pretreating with retinol palmitate effectively protected the heart from myocardial I/R injury through balancing intracellular oxidants and antioxidants (273).

### Coumarin

Coumarin compounds represent an important type of naturally occurring and synthetic oxygen-containing heterocycles with a typical benzopyrone framework. This type of special benzopyrone structure enables its derivatives to readily interact with a diversity of enzymes and receptors in organisms through weak bond interactions, thereby exhibiting wide potentiality as medicinal drugs compounds, inclusive of analgesic, anticoagulant anti-inflammatory, antimicrobial, antineoplastic, antioxidant, and immunomodulatory effects (274).

#### Osthole

Osthole is a compound mainly derived from *Cnidium monnieri (L.) Cusson* and *Angelica pubescens Maxim*., and has been used as tonics and aphrodisiacs in clinical practice of traditional Chinese medicine for many years (275). Modern pharmacological studies demonstrated that it possessed antitumor, anti-hepatic, anti-allergic, anti-inflammatory, anti-apoptotic, and estrogen-like effects (276–278). Wang et al. reported that this compound was beneficial to functional recovery after myocardial I/R injury by increasing SOD, GPx, and CAT activities, and decreasing lipid peroxidation products, MDA, and 4-HNE in the damaged heart tissues. The mechanism behind these effects was related to decreasing the expression of the pro-inflammatory factors, increasing anti-inflammatory cytokines, as well as lowering HMGB1, phosphorylated IκB-α, and NF-κB proteins (279).

#### Esculetin

The natural coumarin compound Esculetin (6,7-dihydroxy coumarin) possesses antioxidant, anti-inflammatory, anti-nociceptive, and anti-tumor activities (280, 281). It also protects against I/R injury. In H/R-stimulated H9c2 cells, esculetin promotes cell viability and reduces lactate dehydrogenase (LDH) release. It also reduces ROS and cell death, following H/R injury through the JAK2/STAT3 pathway (282).

### Others

#### Plantamajoside

Plantamajoside (PMS) is a phenylpropanoid glycoside extracted from *Plantago Asiatica L*. with a long history in food and medical application (283). PMS exhibits anti-inflammatory and antioxidant properties (284, 285). Because of its protective effects against cadmium-induced renal injury and its anti-inflammatory and antifibrotic effects, it has been used to treat many diseases (286, 287). In the *in vitro* I/R model, a previous study investigated the protective effects of PMS on H/R-stimulated oxidative stress, inflammation, and apoptosis in H9c2 cells. PMS attenuated myocardial I/R damage by reducing the inflammatory response, oxidative stress, and apoptosis through Akt/Nrf2/HO-1 and NF-κB signaling pathways (288).

#### Diallyl Trisulfide

Garlic (*Allium sativum* L.) has long before been recognized as beneficial for several diseases. One of its main bioactive compounds is diallyl trisulfide (DATS), also known as allitridin or 4,5,6-trithia-1,8-nonadiene. DATS is a natural, stable, and safe component that attracts H2S donors for *in vivo* studies with an eye to clinical relevance (289). Jeremic et al. reported that DATS consumption could improve heart functions and prevent the oxidative and histoarchitectural variation in the heart suffering from *ex vivo* induced I/R heart injury (290).

#### Eleutheroside E

Eleutheroside E (EE) is a bioactive component of *Eleutherococcus senticosus* (*Rupr. and Maxim*.) *Maxim*. It significantly alleviates physical fatigue and promotes endurance performance, protects against neuritic atrophy and neuron apoptosis, and inhibits inflammatory gene expression (291). Previous studies have shown that treating with EE remarkably limited H/R-induced damage in heart tissue by reducing oxidative stress, inactivating NF-κB, and modulating metabolic responses. Moreover, EE reprograms metabolic action. This evidence proved EE to be potentially valuable in treating H/R-injured heart tissue and emphasized the relationship between EE's protection and metabolic reprogramming (292).

#### Salidroside

Salidroside is a bioactive compound with anti-inflammatory, anti-cancer, anti-oxidant, and anti-fatigue effects (293, 294). A previous study demonstrated that the mechanism of Sal's protective effects against myocardial I/R damage was related to the inhibition of the TLR4/NF-κB signaling pathway, inflammatory response, and cardiomyocyte apoptosis (295).

#### Glycyrrhizin

Glycyrrhizin, one of the most effective ingredients of the root extraction of *Glycyrrhiza glabra L*., is consisted of glucuronic acid and glycyrrhetinic acid and possesses anti-allergic, anti-oxidant, anti-ulcer, anti-viral, anti-cancer, and immunomodulatory effects (296, 297). It also protects the liver and stabilizes the cell membrane. This compound has been broadly used in Europe and the Middle East (298). It was reported that, in rats, glycyrrhizin triggered HMGB1 and the blocked p38 and JNK pathways, ultimately reducing myocardial I/R damage by attenuating oxidative stress, iNOS, and inflammatory reactions *in vivo* (299).

#### Cornuside

Cornuside is a secoiridoid glucoside derived from the fruit of *Cornus officinalis Siebold and Zucc*., which has long been used for attenuating inflammation and promoting blood circulation. It has been found that the crude extract of this fruit had pharmacological effects including, anti-neoplasm, anti-sepsis, antiinflammatory, anti-diabetic nephropathy, and hepatoprotection effects (300, 301). It was reported that in rats suffering from myocardial I/R injury, cornuside decreased infarct volume, improved hemodynamics parameters, and alleviated myocardial injury *via* inhibiting PMN infiltration and MPO activity, decreased pro-inflammatory factors, and reduced phosphorylated IB- and NF-B proteins (302).

### Phytochemicals and Signal Transduction Pathways

**Figure 2:**
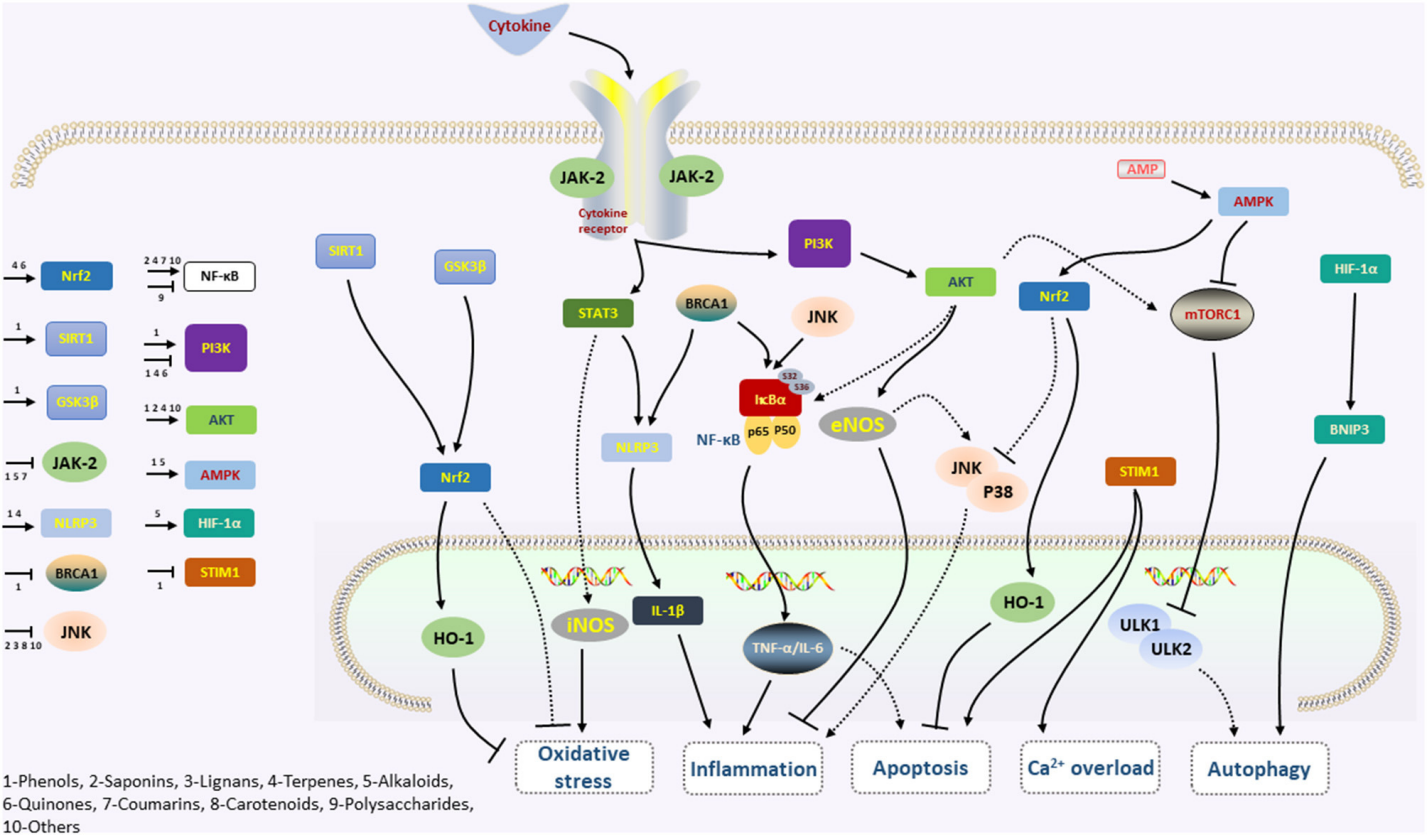
The simplified mechanism scheme of phytochemicals in cardiovascular disease. Phytochemicals reduce the phosphorylation of STAT3 by inhibiting JAK2, which is activated following the binding of cytokines and cognate receptors. Inhibition of the JAK/STAT pathway leads to decreasing of iNOS and NLRP3/IL-1β levels, and thus protects against oxidative stress and inflammation. Activation of the AMPK signaling pathway may also play a key role in the anti-inflammation, further acting on the mTOR and Nrf2 factors and participating in the actions of phytochemicals on oxidative stress, apoptosis, and autophagy. Moreover, the NF-κB signaling pathway, activated by the BRCA1, JNK, and AKT, promotes the expression of TNF-α and IL-6, which regulate inflammation and apoptosis. The phytochemicals are also against apoptosis and inhibit Ca2+ accumulation *via* the STIM1 pathway. The PI3K-AKT signaling pathway is activated by many types of cellular stimuli or toxic insults, activates downstream mTOR, eNOS, and NF-κB, and sequentially regulates the inflammation and apoptosis.

We systematically summarized the phytochemicals' characteristics for their cardioprotective mechanisms in preventing myocardial I/R injury from experimental studies (Table 1). Various internal mechanisms related to myocardial I/R injury that control the fate of cardiomyocytes by phytochemicals interventions are systematically summarized (Figure 2). Among several signal transduction pathways, NF-κB, PI3K/Akt, Nrf2/HO-1, JAK2/STAT, mTOR, and AMPK signaling pathways take an important position in the modulation of myocardial I/R injury by phytochemicals.

**Table 1:** The mechanisms of phytochemicals against myocardial I/R injury from experimental studies.

**Phytochemical name**	**Chemical structure depiction**	**Study model**	**Dose, route, and duration of administration**	**Mechanism**	**References**
**Phenols**					
Paeonol	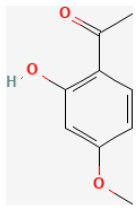	Hypoxia for 2 h/reoxygenation for 2 h in H9c2 cells	10 μmol/L for 18 h beforeH/R	Suppressed oxidative stress, down-regulated inflammatory responses (BRCA1/ROS-regulated NLRP3 inflammasome/IL-1β and NF-κB/TNF-α/IL-6 pathways)	(44)
Oridonin	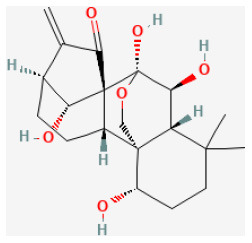	LAD ligation ischemia for 0.5 h/reperfusion for 24 h in C57BL/6 mice	10 mg/kg oral for 7 days before I/R	Suppressed oxidative stress, down-regulated inflammatory responses (NLRP3 inflammasome pathways)	(55)
Baicalin	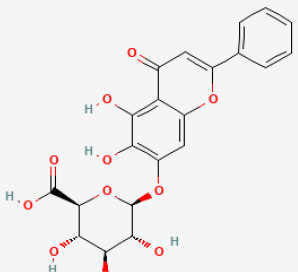	LAD ligation ischemia for 45 min/reperfusion for 180 min in SD rat	20, 60, 120 mg/kg oral for 14 days before I/R	Protected against inflammation through reducing the phosphorylation of JAK2/STAT3 and decreasing the levels of iNOS and IL-1β	(64)
		LAD ligation ischemia for 30 min/reperfusion for 120 min in SD rat / hypoxia for 6 h/reoxygenation for 4 h in Primary rats' cardiomyocytes	100 mg/kg oral for 14 days befor I/R/10 μmol/L for 30 min before H/R	Inhibited apoptosis (CaSR/ERK1/2 signaling pathway)	(63)
		LAD ligation Ischemia for 30 min/reperfusion for 120 min in SD rat	50, 100, 200 mg/kg oral before I/R	Inhibited apoptosis, and inflammation (activated PI3K/Akt but suppressed NF-κB signaling pathways)	(62)
Resveratrol	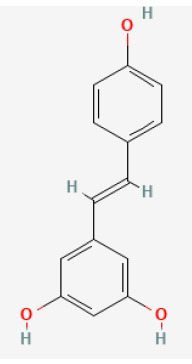	LAD ligation ischemia for 30 min/reperfusion for 120 min in C57BL/6 mice/hypoxia for 3 h/reoxygenation for 3 h in Neonatal rat ventricular cardiomyocytes	50 mg/kg oral for 14 days before I/R/10, 30, 50 μmol/L for 24 h before H/R	Exerted anti-apoptosis and inhibited Ca2+ accumulation (STIM1 pathway)	(71)
		LAD ligation ischemia for 30 min/reperfusion for 120 min in diabetic SD rat	20 mg/kg injection for 7 days before I/R	Inhibited oxidative stress (upregulated SIRT1 and downregulated GSK3β, contributing to improving the expression of Nrf2)	(303)
		LAD ligation ischemia for 30 min/reperfusion for 120 min in SD rat	10 mg/kg oral for 287 days before I/R	Activated autophagy (upregulated Beclin 1/LC3-II), and inhibited inflammatory responses (TNF-α, IL-6)	(34)
		Hypoxia for 24 h/reoxygenation for 24 h in cardiomyocytes	40 μmol/L for 24 h before H/R	Suppressed myocardial apoptosis (inhibited PI3K/AKT/e-NOS pathway)	(72)
6-Gingerol	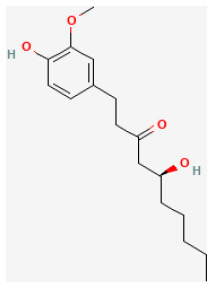	LAD ligation ischemia for 30 min/reperfusion for 120 min in SD rat	6 mg/kg injection for 30 min before I/R	Inhibited cardiomyocyte apoptosis (upregulated the expression of PI3K/Akt signaling pathway)	(85)
Oleuropein	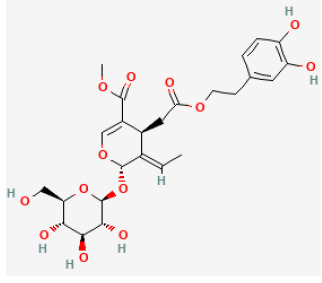	LAD ligation ischemia for 30 min/reperfusion for 180 min in SD rat	20 mg/kg oral for 2 days before I/R	Inhibited apoptosis and inflammation *via* decreasing NF-κB expression and IKB-α degradation pathway	(86)
Calycosin-7-O-β-D-glucoside	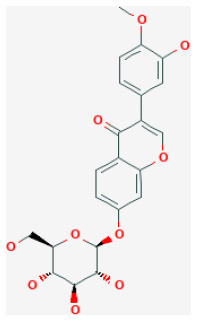	LAD ligation ischemia for 45 min/reperfusion for 180 min in C57BL/6 mice	30 mg/kg oral for 30 min before I/R	Protected against cell apoptosis by activating the JAK2/STAT3 signaling pathway *via* up-regulation of IL-10	(90)
Puerarin	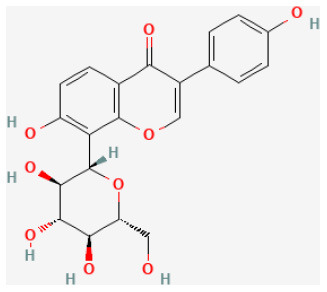	LAD ligation ischemia for 30 min/reperfusion for 180 min in diabetic SD rat	25, 50, 100 mg/kg oral for 28 days before I/R	Suppressed apoptosis, oxidative stress and inflammation (up-regulation of VEGFA/Ang-1 and down-regulation of NF-κB pathways)	(94)
Polydatin	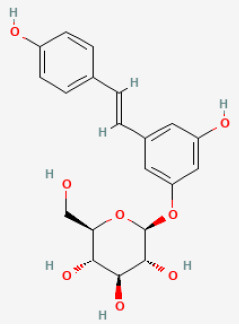	LAC ligation ischemia for 30 min/reperfusion for 120 min in C57BL/6 mice/hypoxia for 3 h/reoxygenation for 3 h in Neonatal rat cardiomyocytes	7.5 mg/kg injection before I/R/1, 10, 100 μmol/L for 1 h before H/R	Reduced ROS and cell death by promoting autophagic flux to clear damaged mitochondria	(76)
Hesperidin	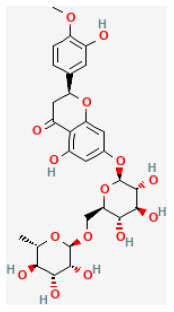	LAD ligation ischemia for 30 min/reperfusion for 240 min in SD rat	200 mg/kg oral for 3 days before I/R	Inhibited excessive autophagy *via* activating the PI3K/Akt/mTOR pathway	(31)
Luteolin	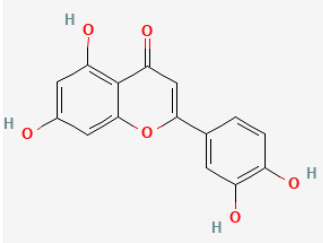	LAD ligation ischemia for 30 min/reperfusion for 24 min in C57BL/6 mice/hypoxia for 2 h/reoxygenation for 2 h in HL-1 cells	25 μg/kg injection for 3 days before I/R/8 μmol/L for 24 h before H/R	Enhanced SERCA2a through SUMOylation at lysine 585 to protect cardiomyocytes	(104)
Honokiol	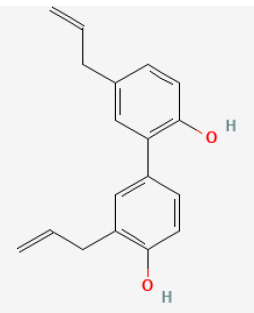	LAD ligation ischemia for 45 min/ reperfusion for 3 h in C57BL/6 mice/hypoxia for 3 h/reoxygenation for 3 h in cardiomyocytes	10 μmol/L injection for 15 min before I/R/5, 10, 20, 40, 80 μmol/L for 3 h before H/R	Promoted autophagic flux (Akt signaling pathway)	(109)
		LAD ligation ischemia for 30 min/ reperfusion for 240 min in diabetic SD rat/hypoxia for 1 h/reoxygenation for 4 h in H9C2 cell	5 mg/kg oral for 7 days before I/R/1,2,5 μmol/L for 2 h before H/R	Ameliorated oxidative damage and apoptosis (SIRT1-Nrf2 signaling pathway)	(108)
Tournefolic acid B	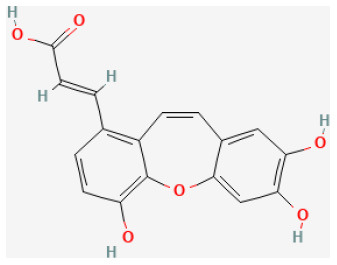	Ischemia for 45 min/reperfusion for 60 min in isolating heart	0.5, 1.2 μg/ml perfusate for 20 min before I/R	Suppressed ER stress, oxidative stress, and apoptosis (enhanced the phosphorylation of PI3K and AKT, inhibited the expression of CHOP and Caspase-12, reduced the phosphorylation of JNK, and increased Bcl-2/Bax ratio)	(110)
Orientin	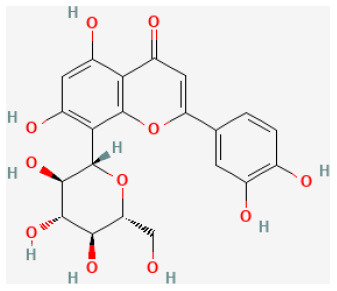	Hypoxia for 12 h/reoxygenation for 12 h in cardiomyocytes	3, 10, 30 μmol/L for 12 h beforeH/R	Promoted autophagy and enhances cell survival (increasing AMPK-mTORC1 signaling pathway and enhancing the interaction of Beclin 1/Bcl-2)	(113)
Icariin	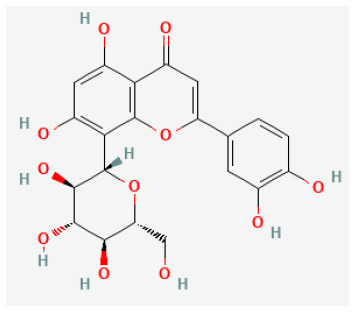	LAD ligation ischemia for 30 min/reperfusion for 120 min in SD rat	10 mg/kg injection after ischemia	Reduced the apoptosis (PI3K/Akt/eNOS pathway)	(120)
Curcumin	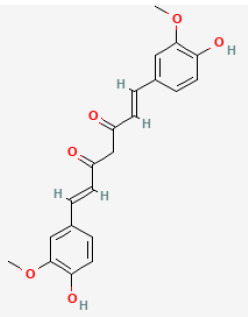	LAD ligation ischemia for 30 min/reperfusion for 180 min in SD rat	10, 20, 30 mg/kg oral for 20 days before I/R	Decreasing oxidative damage and inhibiting myocardium apoptosis (JAK2/STAT3 signal pathway)	(123)
Salvianolic acid A	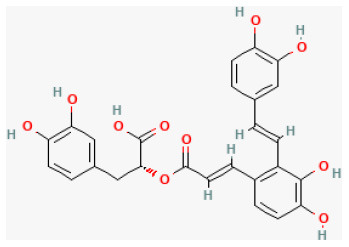	Ischemia for 30 min/reperfusion for 120 min in isolating heart	20 μmol/L before I/R	Exerted an anti-apoptotic effect and improves cardiac function (JNK/PI3K/Akt signaling pathway)	(126)
Astilbin	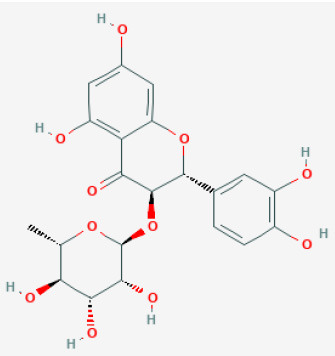	LAD ligation ischemia for 30 min/ reperfusion for 24 h in diabetic rat/hypoxia for 6 h/reoxygenation in H9C2 cell	12.5, 25, 50, 100 mg/kg injection for 4 h befor I/R/1.5, 5, 15, 50 μmol/L for 24 h before H/R	Blocked inflammatory cascade (HMGB1-dependent NF-κB signaling pathway)	(130)
Eupatilin	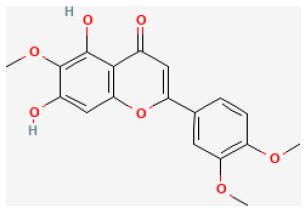	Hypoxia for 3 h/reoxygenation 2 h in H9C2 cell	0.1, 1, 10 μmol/L for 24 h before H/R	Suppressed oxidative stress and apoptosis (Akt/GSK-3β signaling pathway)	(27)
Epigallocatechin-3-gallate	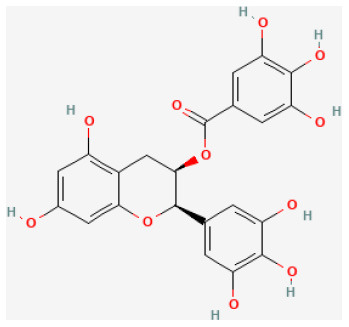	LAD ligation ischemia for 30 min/reperfusion for 120 min in SD rat	10 mg/kg injection after ischemia	Mitigated cell death (activating the RISK pathway and attenuating p38 and JNK)	(142)
Icariin	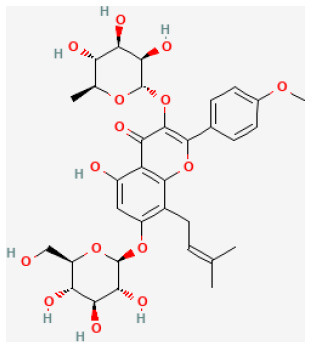	LAD ligation ischemia for 30 min/reperfusion for 120 min in SD rat	10 mg/kg injection for 5 min before I/R	Decreased inflammatory cytokine TNF-α and IL-10, and inhibited apoptosis (PI3K/Akt signaling pathway)	(144)
Troxerutin	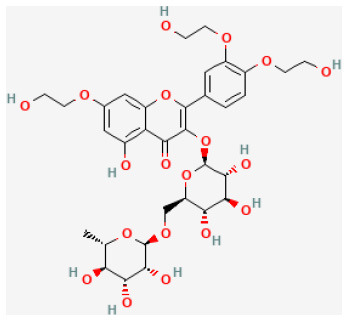	LAD ligation ischemia for 30 min/reperfusion for 60 min in isolating heart	150 mg/kg oral for 1 month before I/R	Exerted significant anti-arrhythmic and anti-inflammatory effects because of the inhibition of inflammatory cytokines activity and reduction of inflammatory reactions	(147)
Isoquercitrin	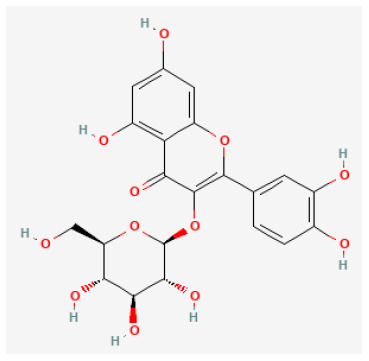	Hypoxia for 6 h/reoxygenation for 12 h in H9C2 cell	20, 40, 80 mg/ml for 24 h before H/R	Inhibited apoptosis and ROS generation by protecting mitochondrial function and preventing cytochrome c release	(153)
Silibinin	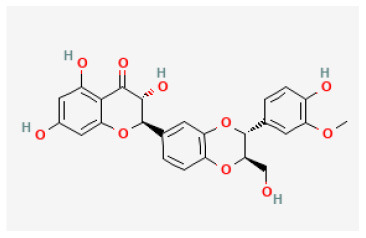	LAD ligation ischemia for 30 min/reperfusion for 24 h in C57BL/6 mice/hypoxia for 6 h/reoxygenation in H9C2 cell	100 mg/kg injection for 7 days before I/R	Inhibited cardiomyocytes apoptosis, reduced ER stress and oxidative stress, and modulating inflammatory response *via* deactivation of NF-κB signaling pathway.	(170)
**Saponins**					
Polyphyllin I	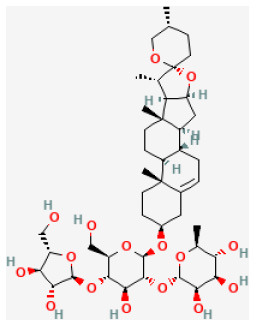	LAD ligation ischemia for 30 min/reperfusion for 120 min in SD rat	150 mg/kg injection for 2 weeks before I/R	Inhibiting inflammatory response and oxidative stress (NF-κBp65 signaling pathway)	(176)
Ginsenoside Rb1	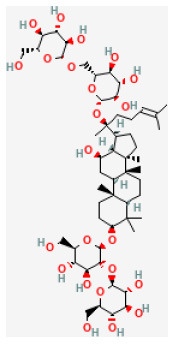	LAD ligation ischemia for 45 min/reperfusion for 120 min in SD rat	20, 40, 80 mg/kg injection for 3 days before I/R	Decreased the expression of apoptotic related proteins e.g., cleaved-caspase 3 (mTOR signaling pathway)	(25)
Gypenoside A	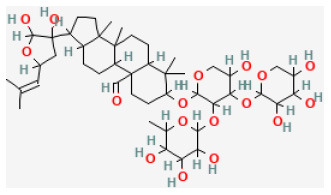	Hypoxia for 2 h/reoxygenation for 24 h in H9C2 cell	20 μmol/L for 24 h before H/R	Suppressed miR-143-3p *via* the activation of AMPK signaling	(183)
Ginsenoside Rg3	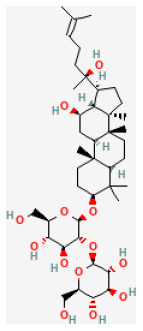	LAD ligation ischemia for 30 min/reperfusion for 24 h in SD rat	5, 20 mg/kg oral for 7 days before I/R	Attenuated apoptosis and inflammation	(192)
Ginsenoside Rb3	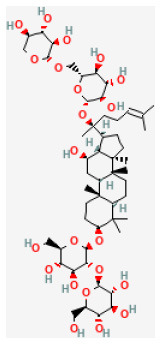	Hypoxia for 4 h/reoxygenation for 24 h in H9C2 cell	2, 5 μmol/L for 24 h before H/R	Inhibited apoptosis (JNK/NF-κB activation signaling pathway)	(194)
Platycodin D	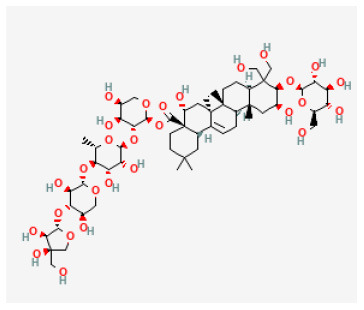	Hypoxia for 4 h/reoxygenation for 24 h in H9C2 cell	5, 10, 20, 40 μmol/L for 24 h before H/R	Inhibited oxidative stress and apoptosis (Inducing the activation of Akt/Nrf2/HO-1 pathway)	(197)
**Lignans**					
Isovaleroylbinankadsurin A	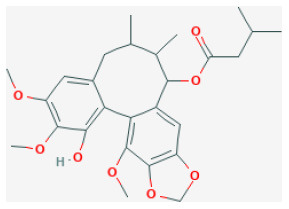	LAD ligation ischemia for 45 min/reperfusion for 120 min in C57BL/6 mice/hypoxia for 150 min/reoxygenation for 60 min in neonatal rat ventricle myocytes and H9C2 cell	10, 20, 40 mg/kg injection for 1 h before I/R/0.3, 1, 3 μmol/L for 1 h before H/R	Blocked the apoptosis and inhibiting the ROS generation (activating GR dependent RISK pathway)	(202)
Sauchinone	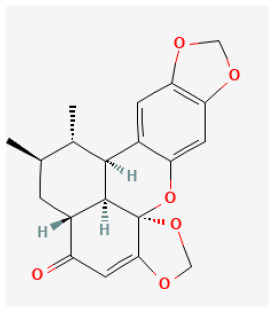	LAD ligation ischemia for 30 min/reperfusion for 2 h in isolating heart	10 mg/kg injection for 30 min before I/R	Exerted anti-inflammatory and antioxidant effects through inhibition of phosphorylation of p38 and JNK death signaling pathways	(207)
**Terpenes**					
Glaucocalyxin A	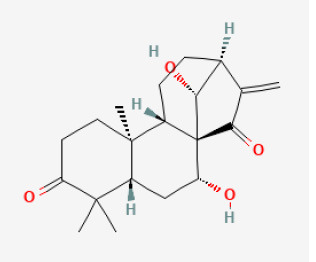	Ischemia for 1 h/reperfusion for 24 h in C57BL/6J mice	10 mg/kg injection after ischemia	Reducted microvascular thrombosis	(212)
		Hypoxia for 24 h/reoxygenation for 2 h in H9c2 cells	5, 10, 20, and 40 μmol/L for 2 h before H/R	Suppressed apoptosis and oxidative stress (Akt/Nrf2/HO-1 signaling pathway)	(213)
Artemisinin	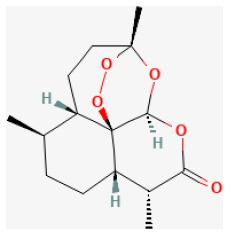	LAD ligation ischemia for 0.5 h/reperfusion for 2 h in SD rat	14 mg/kg oral for 2 weeks before I/R	Suppressed NLRP3 inflammasome activation (decreasing NLRP3, ASC, cleaved caspase-1, IL-1β)	(47)
Geniposide	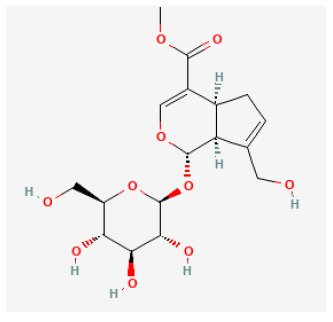	LAD ligation ischemia for 0.5 h/reperfusion for 2 h in SD rat/hypoxia for 12 h/reoxygenation for 4 h in H9c2 cells	100 mg/kg oral 30 min before I/R/40 μmol/L for 30 min before H/R	Inhibited the expression of autophagy-related proteins and autophagosome accumulation (activating AKT/mTOR signaling pathways)	(217)
Ginkgolide B	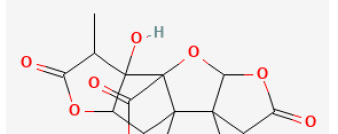	Ischemia for 1 h/reperfusion for 1 h in SD rat	15 mg/kg injection for 10 min before ischemia	Inhibited ER stress-induced apoptosis *via* PI3K/AKT/mTOR signaling pathway	(224)
		Ischemia for 40 min/reperfusion for 120 min in SD rat	8, 16, 32 mg/kg injection for 7 days before ischemia	Alleviated inflammatory response (inhibiting NF-κB p65 subunit translocation, IκB-α phosphorylation, IKK-β activity, as well as the downstream inflammatory cytokines and proteins expressions *via* zinc finger protein *A20*)	(223)
Triptolide	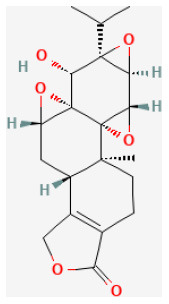	LAD ligation ischemia for 45 min/reperfusion for 3 h in Wistar rat	25, 50, 100 μg/kg injection for 12 h before I/R	Reduced inflammation and oxidative stress (Nrf2/HO-1 defense pathway)	(230)
**Alkaloids**					
Berberine	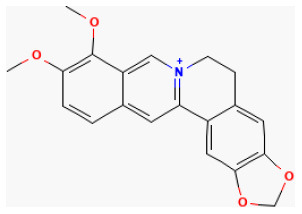	LAD ligation ischemia for 30 min/reperfusion for 120 min in Wistar rat/hypoxia for 4 h/reoxygenation for 3 h in H9C2 cell	300 mg/kg oral for 3 days before I/R/50 μmol/L for 3 h before H/R	Promoted mitochondrial autophagy, reduced myocardial enzyme activity, induced cardiomyocytes proliferation, inhibited cardiomyocytes apoptosis (HIF-1α/BNIP3 pathway)	(236)
Galanthamine	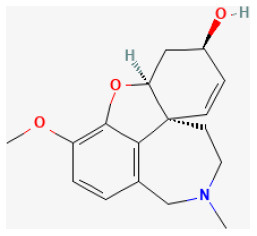	LAD ligation ischemia for 30 min/reperfusion for 120 min in SD rat	1, 3 mg/kg injection for 30 min before I/R	Prevented endoplasmic reticulum stress-related apoptosis, and myocardial fibrosis *via* promoting AMPK and Nrf2-related proteins (AMPKα1, Nrf2 and HO-1)	(239)
Matrine	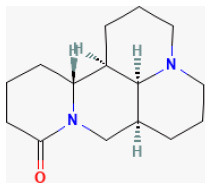	LAD ligation ischemia for 30 min/reperfusion for 24 h in SD rat/hypoxia for 4 h/reoxygenation for 6 h in cardiomyocytes	50, 100 mg/kg injection before I/R/200, 400 μmol/L after hypoxia	Decreased lactate dehydrogenase release, creatine kinase activity, and cardiomyocytes apoptosis (JAK2/STAT3 signaling pathway)	(244)
Capsaicin	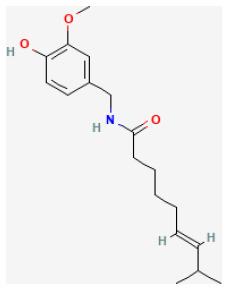	Hypoxia for 3 h/reoxygenation for 3 h in H9C2 cell	5, 10, 20, 40, 80 μmol/L for 36 h before H/R	Attenuated generation of ROS, inhibited mPTP opening and caspase-3 activation, downregulated Bax, upregulated 14-3-3η and Bcl-2, and ultimately reduced apoptosis	(250)
**Quinones**					
Sodium tanshinone IIA sulfonate	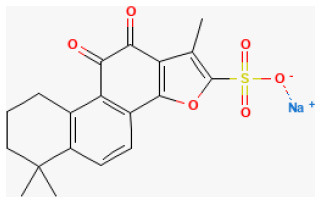	LAD ligation ischemia for 30 min/reperfusion for 24 h in SD rat	8 mg/kg injection for 15 min before ischemia and for 0.5, 1, 2, 4, 6 h after ischemia	Protected against oxidative stress and inflammatory responses (NF-κB/HO-1 signaling pathway)	(258)
Shikonin	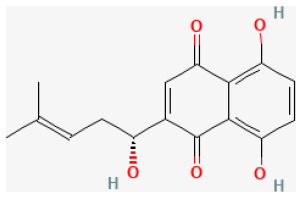	Hypoxia for 12 h/reoxygenation for 24 h in H9c2 cells	10, 20, 40 μmol/L for 48 h before H/R	Suppressed apoptosis and increased cell viability, attenuated LDH release (PI3K/Akt signaling pathway)	(261)
**Polysaccharides**					
Fucoidan	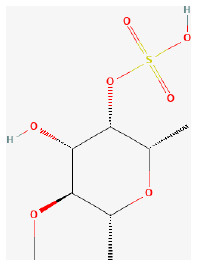	LAD ligation ischemia for 30 min/reperfusion for 0.5–6 h in Wistar rat	27 μg/kg/min injection from 10 min before to 6 h after reperfusion	Blockaded of P-selectin-mediated neutrophil rolling on the vessel wall	(264)
		LAD ligation ischemia for 30 min/reperfusion for 2 h in SD rat	50, 100, 200 mg/kg oral for 7 days before I/R	Regulated the inflammation response *via* HMGB1 and NF-κB inactivation in I/R-induced myocardial damage	(265)
**Carotenoids**					
Lycopene	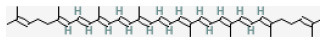	LAD ligation ischemia for 20 min/reperfusion for 40 min in C57BL/6 mice/hypoxia for 2 h/reoxygenation for 2 h in HL-1 cells	1 μmol/L injection after ischemia/1, 2, 4 μmol/L for 2 hafter H/R	Inhibited ROS accumulation and inflammation (JNK signaling pathway)	(270)
Retinol palmitate	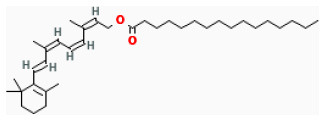	LAD ligation ischemia for 40 min/reperfusion for 4 h in C57BL/6 mice/hypoxia for 2 h/reoxygenation for 4 h in H9C2 cells	12, 36 mg/kg injection for 3 days before I/R/0.1, 1 μmol/L for 4 h before H/R	Inhibited oxidative stress and apoptosis	(273)
**Coumarin**					
osthole	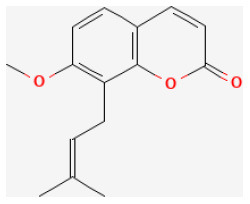	LAD ligation ischemia for 30 min/reperfusion for 24 h in SD rat	1, 10, 50 mg/kg injection before I/R	Exerted antioxidant and anti-inflammatory effect (inhibiting the expression of HMGB1 and IκB-α/NF-κB signaling pathway)	(279)
Esculetin	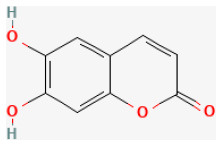	Hypoxia for 3 h/reoxygenation for 6 h in H9c2 cells	5, 10, 20, 40 μmol/L for 24 h before H/R	Suppressed oxidative stress and apoptosis (JAK2/STAT3 signaling pathway)	(282)
**Others**					
Plantamajoside	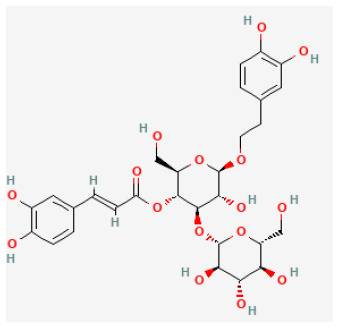	Hypoxia for 6 h/reoxygenation for 12 h in H9c2 cells	10, 20, 40, and 80 μmol/L for 24 h before H/R	Suppressed inflammation and oxidative stress (Akt/Nrf2/HO-1 and NF-κB signaling pathways)	(288)
Diallyl trisulfide	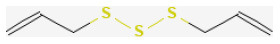	Ischemia for 30 min/reperfusion for 1 h in isolating heart	40 mg/kg oral for 3 weeks before I/R	Suppressed oxidative stress and apoptosis with increasing relative gene expression of eNOS, SOD-1 and−2, Bcl-2 and decreasing relative gene expression of NF-κB, IL-17A, Bax, and caspases-3 and−9	(290)
Eleutheroside E	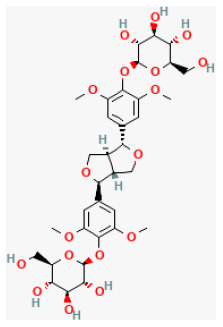	Hypoxia for 4 h/reoxygenation for 24 h in H9c2 cells	30, 60, and 100 μmol/L for 3 h before H/R	Reduced oxidative stress (NF-κB signaling pathway)	(292)
Salidroside	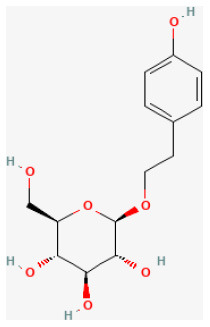	Ischemia for 30 min/reperfusion for 24 h in SD rat	20, 40 mg/kg oral for 7 days before I/R	Suppressed inflammation and apoptosis (TLR4/NF-κB signaling pathway)	(295)
Glycyrrhizin	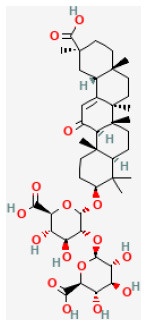	Ischemia for 30 min/reperfusion for 24 h in SD rat	0, 2, 4, 10 mg/kg injection for 30 min before I/R	Reduced oxidative stress, iNOS and inflammatory reactions (blocked p38 and JNK signaling pathway)	(299)

### NF-κB Signaling Pathway

NF-κB (Nuclear Factor-kappa B) is composed of different transcription factors—the Rel family. The Rel/NF-κB family regulates immune and inflammatory responses. Activated NF-κB prevents ischemic injury and inhibits both inflammation and apoptosis (304). Paeonol significantly alleviates hypoxia and attenuates I/R injury in H9C2 cells through the BRCA1/ROS-regulated NF-κB/TNF-α/IL-6 pathways and NLRP3 inflammasome (44). Puerarin exerts a similar effect by suppressing NF-κB and upregulating VEGFA/Ang-1 in diabetic rats with myocardial I/R injury (94). Similarly, fisetin reduces ischemic injury and oxidative damage by inhibiting cytokines, such as IL-1β and TNF-α (305). Polyphenols modulate the immune system by inhibiting NF-κB (304).

### PI3K/Akt Signaling Pathway

PI3K and the downstream target serine/threonine kinase Akt are crucial in various physiological processes. Activated PI3K/Akt signaling pathways is protective in myocardial I/R injury (306, 307). A study by Wang et al. suggests it is associated with H/R-induced cardiomyocyte apoptosis in Shikonin pretreated cells (261). Another study shows resveratrol inhibits I/R injury-induced cardiomyocyte apoptosis by regulating phosphorylation levels of PI3K/Akt/e-NOS pathway-related proteins (72). The PI3K/Akt signaling pathway regulates the life cycle of cardiomyocytes by regulating the morphology and function (308). 6-Gingerol possesses similar potent *via* this pathway (85).

### Nrf2/HO-1 Signaling Pathway

Normally, Nrf2is a transcription factor that regulates the expression of several factors involved in the cellular defense against oxidative stress and inflammation, including heme oxygenase-1 (HO-1) (309). Once activated, it is stabilized and translocates to the nucleus, and binds antioxidant response element (ARE), which activates HO-1 (310). Numerous studies have shown the potential role of the Nrf2/HO-1 pathway in myocardial I/R injury (311). A study by Yu et al. found Nrf2 accumulated more in the nuclear due to triptolide in reperfused myocardium (230). Also, triptolide promoted the activity and expression of HO-1. This study proved triptolide was cardioprotective by activating the Nrf2/HO-1 defense pathway in treatments in I/R injuries (312). In addition, Zhou et al. demonstrated 160-nM triptolide pretreatment for a short period (<6 h) raised the levels of nuclear Nrf2 and HO-1 in H9c2 cardiomyocytes, but they are downregulated if pretreatment lasted for a longer period (> 9 h) (313). Glaucocalyxin A is also reported to increase cell viability and decrease oxidative stress in H9c2 cells, resulting in fewer cell death from H/R-stimulated oxidative damage. The protective effect of GLA is proved to be associated with the activation of the Akt/Nrf2/HO-1 signaling pathway (213).

### JAK2/STAT Signaling Pathway

Several reports proposed that JAK/STAT signaling is associated with cardiac dysfunction in myocardial I/R injury (314). JAKs are rapidly recruited to the receptor and activated after the upstream receptor molecule, and then catalyze its tyrosine phosphorylation. This process supplies binding sites for the SH2 domain of STATs, ultimately leading to specific gene transcription. In particular, myocardial I/R injury activated JAK1, and JAK2, in turn, activates STAT1 and STAT3. STAT1 promotes apoptosis, while STAT3 protects cardiomyocyte (315). Ming Xu reported baicalin alleviated post-I/R myocardial injury and reduced inflammation *via* JAK/STAT pathway (64). CG pretreatment protected the myocardium against I/R injury by upregulating IL-10 expression (90). Matrine can attenuate myocardial I/R injury by upregulating HSP70, which can be activated by the JAK/STAT pathway (244).

### MTOR Signaling Pathway

mTOR is a mammalian target of rapamycin (RAPA) and downregulates autophagy (316). Luo et al. found that GP upregulated p-mTOR^Ser2448^ expression and inhibited autophagy, but these effects were counteracted by RAPA. They also observed that RAPA enhanced p-AKT^Ser473^ expression, which might be associated with the activation of upstream AKT by mTOR inhibition (217). However, RAPA's effects on activating autophagy were inconsistent in myocardial I/R injury. In myocardial I/R injury, GRb1's effects are also controversial. Some studies have shown that mTOR switched on I/R (317), whereas others tend to hold the opposite view. Li et al. proved p-mTOR to be in an inhibitory state in I/R injury. Remarkably, GRb1 treatment reversed the inhibitory state and activated it (25). P-mTOR changes are dynamic after myocardial cell injury, and this may account for the difference in the performance of mTOR in I/R across studies.

### AMPK Signaling Pathway

AMPK regulates cell homeostasis and reprograms metabolism. Hou et al. reported Gal alleviated I/R-induced cardiac dysfunction, reduced ERS-related apoptosis, and inhibited myocardial fibrosis by suppressing AMPK/Nrf2 pathways (239). The relationship between the cardio-protective effect of GP depends on suppressing miR-143-3p *via* activating AMPK, which furthered the understanding by connecting their function with miRs (183).

## Conclusion and Perspectives

To date, this review provides the most comprehensive overview of the current knowledge of phytochemicals that interfere with the myocardial I/R injury. Among the phytochemicals with potential anti-I/R injury ability, phenolic compounds take up the largest proportion (45.1%). Saponins, lignans, terpenes, alkaloids, quinones, coumarin, carotenoids, and other compounds make up the remainder, respectively. In addition, phytochemicals extensively modulated autophagy, oxidative stress, Ca^2+^ overload, apoptosis, inflammation, and key regulatory targets and proteases activities. From this point of view, phytochemicals may be a potential panacea for myocardial I/R injury treatment, and studies on their mechanisms rule out the possibility of applying a single molecule as a pathophysiological cause of myocardial I/R injury, while most natural products have more than one “target” and may affect multiple pathways.

Although phytochemicals found in natural products have made great progress in alleviating myocardial I/R injury, future studies focusing on human clinical trials of several potent phytochemicals and their combinations should be carried out. Theoretically, animal models help to explore the probable mechanism; however, there is still a huge anatomic and/or physiological gap between the different species, which may possibly be responsible for the inconsistency between preclinical studies and clinical studies currently (70). Therefore, more appropriate experimental models and precise pharmaceutical intervention studies are needed to simulate human heart physiology. Furthermore, phytochemicals must be investigated for the risk assessment and safety evaluation to observe any undesirable effects, which may hinder further use of phytochemicals as a cardioprotective adjuvant in the human body, as well as the enthusiasm for further pharmaceutical development. In addition, there may be a paradox that the cardio protection of phytochemicals is associated with inhibition of cell death, but it is an antineoplastic activity with the promotion of cell death (318). Cancer cells express different levels of apoptosis-promoting or inhibiting proteases compared to cardiomyocytes, which might partly explain these differences (318). Overall, phytochemicals may be a potential panacea for myocardial I/R injury treatment, but more research is needed to support this promising means of enhancing prognosis and, possibly, prevention.

## Author Contributions

QL and JW contributed to study conception and design, contributed to final approval, and overall responsibility for this published work. CC, L-TY, and B-RC contributed to acquisition, analysis, and interpretation of data. J-LX, YC, J-LJ, R-LF, LX, X-YQ, DL, JL, YL, X-YC, J-JL, and KZ contributed to article revision. All the authors contributed to the article and approved the submitted version.

## Funding

This work was supported by the Fundamental Research Funds for the Central Universities (No. 2019-JYB-TD-008), the National Natural Science Foundation of China (No. 81803906), and the special project of Research and Demonstration Application of Clinical Diagnosis and Treatment Technology of Beijing Science and Technology Plan (No. Z1910000006619070).

## Conflict of Interest

The authors declare that the research was conducted in the absence of any commercial or financial relationships that could be construed as a potential conflict of interest.

## Publisher's Note

All claims expressed in this article are solely those of the authors and do not necessarily represent those of their affiliated organizations, or those of the publisher, the editors and the reviewers. Any product that may be evaluated in this article, or claim that may be made by its manufacturer, is not guaranteed or endorsed by the publisher.

## Abbreviations

AMI: acute myocardial infarction
STEMI: ST-elevation myocardial infarction
pPCI: primary percutaneous coronary intervention
IS: infarct size
I/R: ischemia-reperfusion
NADH+: nicotinamide adenine dinucleotide
ROS: reactive oxygen species
STAT3: signal transducer and activator of transcription 3
BCL-C: B-cell lymphoma-C
Bcl-xL: B-cell lymphoma-xL
mTOR: mammalian target of rapamycin
Cyt c: cytochrome c
Bcl-2: B-cell lymphoma-2
Bax: BCL2-associated X
H/R: hypoxia-reoxygenation
VSMC: vascular smooth muscle cell
3-MA: 3-methyladenine
HMGB1: high-mobility group box protein 1
GSK-3β: glycogen synthase kinase-3β
PI3K: phosphatidylinositol-3-OH kinase
Akt: protein kinase b
ATP: adenosine triphosphate
NF-κB: nuclear factor kappa-light-chain-enhancer of activated B cells
BRCA1: breast cancer type 1 sensitive protein
NLRP3: nod-like receptor protein 3
TNF-α: tumor necrosis factor-α
IL-6: interleukin-6
Nrf2: nuclear factor-erythroid 2-related factor 2
STIM1: stromal interaction molecule1
Sal-B: salvianolatic acid B
6-G: 6-Gingerol
CK-MB: creatine kinase-MB
LDH: lactate dehydrogenase
ERK: p-extracellular signal-regulated protein kinase
MEK: (p)-mitogen-activated protein kinase kinase
CG: Calycosin-7-O-β-D-glucoside
NO: nitric oxide
HKL: honokiol
TAB: tournefolic acid B
ER: endoplasmic reticulum
AMPK: adenosine monophosphate-activated protein kinase
JAK2: Janus kinase 2
SalA: salvinolic acid A
SA: salvinolic acids
EGCG: epigallocatechin-3-gallate
JNK: c-Jun N-terminal kinases
ERK: extracellular signal-regulated kinase
BCF: bauhinia championii flavone
GAS: gastrodin
AS-IV: astragaloside IV
ISBA: isovaleroylbinankadsurin A
RISK: reperfusion injury salvage kinase
GLA: glaucocalyxin A
GB: ginkgolide B
BBR: berberine
ERS: endoplasmic reticulum stress
SOD: superoxide dismutase
HSP70: heat shock proteins 70
PMS: plantamajoside
DATS: diallyl trisulfide
EE: eleutheroside E
TLR4: toll-like receptor 4
RAPA: rapamycin
SHR: spontaneous hypertension rat
TCM: traditional Chinese medicine.
